# Wearables-derived risk score for unintrusive detection of α-synuclein aggregation or dopaminergic deficit

**DOI:** 10.1016/j.ebiom.2025.105782

**Published:** 2025-06-06

**Authors:** Ann-Kathrin Schalkamp, Kathryn J. Peall, Neil A. Harrison, Valentina Escott-Price, Payam Barnaghi, Cynthia Sandor

**Affiliations:** a Division of Psychological Medicine and Clinical Neuroscience, School of Medicine, Cardiff University, Cardiff, United Kingdom; b UK Dementia Research Institute, Cardiff University, Cardiff, United Kingdom; c Neuroscience and Mental Health Innovation Institute, Division of Psychological Medicine and Clinical Neurosciences, Cardiff, United Kingdom; d Cardiff University Brain Research Imaging Centre (CUBRIC), Cardiff, United Kingdom; e Department of Brain Sciences, Faculty of Medicine, Imperial College London, London, United Kingdom; f UK Dementia Research Institute, Care, Research and Technology, London, United Kingdom; g Bakar Computational Health Sciences Institute, University of California San Francisco, USA; h UK Dementia Research Institute at Imperial College London, United Kingdom

**Keywords:** Parkinson's disease, Smartwatch, Prodromal, Risk modelling

## Abstract

**Background:**

Smartwatch data has been found to identify Parkinson's disease (PD) several years before the clinical diagnosis. However, it has not been assessed against the gold standard but costly and invasive biological and pathological markers for PD. These include dopaminergic imaging (DaTscan) and cerebrospinal fluid alpha-synuclein seed amplification assay (SAA), which are being studied as markers thought to represent the onset of PD pathology.

**Methods:**

Here, we combined clinical and biological data from the Parkinson's Progression Marker Initiative (PPMI) cohort with long-term (mean: 485 days) at-home digital monitoring data collected using the Verily Study Watch. We derived a digital risk score based on sleep, vital signs, and physical activity features to distinguish between PD (N = 143) and healthy controls (N = 34), achieving an area under precision-recall curve of 0.96 ± 0.01. We compared it with the Movement Disorder Society (MDS) research criteria for prodromal PD to detect dopaminergic deficit or α-synuclein aggregation in an at-risk cohort consisting of people with genetic markers or prodromal symptoms without a diagnosis of PD (N = 109, mean age = 64.62 ± 6.86, 40 men and 69 women).

**Findings:**

The digital risk correlated with the MDS research criteria (r = 0.36, p-value = 1.46 × 10^−4^) and was increased in individuals with subthreshold Parkinsonism (p-value = 4.99 × 10^−6^) and hyposmia (p-value = 3.77 × 10^−2^). The digital risk was correlated to a stronger degree with DaTscan putamen binding ratio (r = −0.32, p-value = 6.64 × 10^−4^) than the MDS criteria (r = −0.19, p-value = 6.81 × 10^−3^) but to a weaker degree with SAA (r = 0.2, p-value = 3.9 × 10^−2^) than the MDS (r = 0.43, p-value = 1.3 × 10^−5^). The digital risk score achieved higher sensitivity in identifying synucleinopathy or neurodegeneration (0.59) than the MDS score (0.35) but performed on-par with hyposmia (0.59) with a combination of hyposmia and digital risk score achieving the highest sensitivity (0.71). The digital risk score showed lower precision (0.18) than other models.

**Interpretation:**

A digital risk score from smartwatch data should be further explored as a possible first sensitive screening tool for presence of α-synuclein aggregation or dopaminergic deficit followed by subsequent more specific tests to reduce false positives.

**Funding:**

This project is funded by Welsh Government through 10.13039/100012068Health and Care Research Wales, 10.13039/501100000265Medical Research Council (MRC), 10.13039/501100000383Higher Education Funding Council for Wales, 10.13039/501100017510UK Dementia Research Institute, Alzheimer's Society and Alzheimer's Research UK, Dementia Platforms UK, 10.13039/501100000266UKRI Engineering and Physical Sciences Research Council (EPSRC), 10.13039/501100013342NIHR Imperial Biomedical Research Centre (BRC), 10.13039/501100001279Great Ormond Street Hospital and the 10.13039/501100000287Royal Academy of Engineering, Edmond J. Safra Foundation, Ser Cymru II programme, and the European Regional Development Fund.

**Research in context**

**Evidence before this study**

We searched PubMed with the search term: (“Parkinson” AND “prodromal”) AND (“digital” OR “wearable” OR “smartwatch”) AND (“DaTscan” OR “seed amplification assay”) for articles published in English on or before June 10, 2024, in any field. This resulted in no found articles. However, research into digital markers for early detection of Parkinson's disease ((“Parkinson” AND “prodromal”) AND (“digital” OR “wearable” OR “smartwatch”)) has gained interest over the past years with 44 studies identified of which multiple highlight the potential value of such technology for early screening. Research for biological markers of Parkinson's disease ((“Parkinson” AND “prodromal”) AND (“DaTscan” OR “seed amplification assay”)) has led to two recent publications discussing potential biological definitions of Parkinson's disease of which both include dopaminergic imaging and alpha-synuclein SAA as potential tests. The combination of both these fields has not yet been explored.

**Added value of this study**

This study relates digital risk to biological and pathological markers of Parkinson's disease in an at-risk cohort. The strengths of our analysis include the quantitative evaluation of various risk markers in a well-studied cohort. Key findings in this study include: the digital risk score being elevated in individuals with subthreshold Parkinsonism and hyposmia, the correlation of the digital risk score with not only an established prodromal score (MDS) but also biological and pathological markers, and a higher sensitivity in identifying individuals with biological or pathological markers for the digital risk score than the established MDS criteria.

**Implications of all the available evidence**

Our results show that digital risk scores are related to biological and pathological markers and suggest a crucial role for digital risk scores in early screening for Parkinson's disease, especially as a first indicator in a sequential screening process.

## Introduction

The diagnosis of Parkinson's disease (PD) continues to rely on clinical judgement, requiring evidence of motor signs. However, by that time 50–70% of the neurons producing dopamine, which help control movement, have already degenerated.^1^ Therefore, identifying people prior to this is of high clinical value, and essential for the investigation of neuroprotective therapies. The phase predating the clinical diagnosis is termed ‘prodromal’, can span multiple years, and is characterised by a multitude of symptoms and signs including rapid-eye movement (REM) behavioural sleep disorder (RBD), hyposmia, constipation, or mood disorders.^2^

Existing risk scores trying to identify individuals during the prodromal phase, such as the Movement Disorder Society (MDS) research criteria^3^ or PREDICT-PD,^4^ are based on lifestyle and genetic factors as well as prodromal symptoms. Such prodromal risk scores, however, show low sensitivity over ten-year follow-up (35%).^5^^,^^6^ Biological and pathological markers for PD have shown promising performance in prodromal cohorts, with dopamine transporter (DaT) binding found to be reduced in ∼40% of patients with idiopathic RBD (iRBD)^7^^,^^8^ with 36.48% converting to PD within 4.7 years.^9^ Recently, a cerebrospinal fluid (CSF) test detecting abnormal α-synuclein protein accumulation (seed amplification assay (SAA)) has been found to be highly predictive for idiopathic PD, and to be present in individuals with hyposmia (88.9%) and RBD (84.8%).^10^ Based on DaTscan positivity and α-synuclein SAA, a biological definition and a staging system of PD have been suggested.^11^^,^^12^ Despite the high specificity of the biological and pathological markers, they are not suited for population-based screening due to their associated cost, invasiveness, and time requirements.

Previously, we have shown that one week of accelerometer data can identify people years prior to their clinical diagnosis.^13^ Digital sensor data can be passively collected at home with low-cost devices, addressing the limitations of the above-mentioned markers. The relationship between biological and pathological markers of PD and digital risk has not yet been investigated, with positive findings underlining the validity of digital screening.

In this study, we used data from participants at-risk of developing PD enrolled in the well-characterised Parkinson's disease Progression Marker Initiative (PPMI).^14^ We analysed data gathered over 1.3 years from multi-sensor smartwatches to develop a digital risk score for PD risk. Furthermore, we evaluated this digital risk score by comparing it with existing prodromal, biological, and pathological markers in an unseen at-risk group.

## Methods

### Study cohort

PPMI has collected data from individuals recently diagnosed with PD, individuals at risk, and individuals without a diagnosis since 2010. We focused on individuals who have been supplied with a Verily Study Watch (developed by Verily Life Sciences, FDA-cleared Class II medical device, 510(k) K182456 and K213357), a multi-sensor smartwatch that is equipped with accelerometer, gyroscope, electrocardiogram, and photoplethysmography (Table 1). We used the analytic dataset cohort assignment which was derived by the Consensus Cohort Committee that reevaluated the clinical data of subjects. The at-risk group was formed of people with polysomnography (PSG)-proven RBD, confirmed hyposmia by the consensus committee, or mutations in Mendelian inherited genes considered causative or contributing to increased risk in PD (e.g., *LRRK2, GBA, SNCA, Parkin, Pink1*) (Table 2).

**Table 1:** Derived digital markers as provided by Verily.

Modality	Category	Sensors	#Features	Features	Model
Physical activity	Ambulatory	3-axis accelerometer	1	Hourly walking minutes	2-class classifier (walk/run vs other) trained on 215,000 h of self-report labelled free-living data from 1800 adult subjects with out-of sample performance of 87%
	Step	3-axis accelerometer	1	Hourly step count	Frequency-based model validated against ankle-worn gait monitor on 329 days of free-living data of 75 adult subjects with 18% mean absolute error^15^
Sleep	Sleep onset/offset	Accelerometer, PPG	4	Sleep efficiency, number of awakenings, total sleep time, wake after sleep onset	Algorithm trained on PPG and ECG validated against majority vote of three wearables on 176 nights in home setting of 50 adult subjects with median absolute error of sleep onset of 6 min and 9 min for sleep offset
	Sleep stages	Accelerometer, PPG	4	REM, NREM, light NREM, deep NREM	Algorithm trained on PPG and ECG validated against majority vote of three wearables on 176 nights in home setting of 50 adult subjects with an overall accuracy of 70%^16^
Vital signs	Pulse rate	PPG	1	Total mean pulse rate per hour	Algorithm from ADI validated against heart rate of ECG on one to 2 h of in-clinic data of 50 adult subjects with a mean absolute error of 10.7 beats per minute ADI2023^17^

The different hourly statistics as derived from the smartwatch data are described. This information is taken from the accompanying documents on PPMI LONI. PPG: Photoplethysmography, ECG: Electrocardiography, RMSSD: root mean square of successive differences between normal heartbeats, REM: rapid eye movement.

**Table 2:** Study cohort.

Diagnosis	Subgroup	Sample size	Male sex	Age accelerometry	
			Proportion	Mean	Std
HC	All	35	0.54	66.69	12.23
PD	All	149	0.60	68.48	8.62
At-risk	All	151	0.37	64.49	6.97
At-risk	LRRK2	59	0.41	64.60	6.93
At-risk	GBA	90	0.34	63.91	7.06
At-risk	Hyposmia	29	0.48	68.57	7.34
At-risk	RBD	2	1.00	70.40	4.68
At-risk	Positive DaTscan	14	0.50	70.28	3.62
At-risk	Positive SAA	19	0.53	69.98	5.78

Demographic and prodromal marker information for the PD, healthy control, and the different at-risk groups.

### Digital data

Digital data collection took place between 2018 and 2020 inviting all US-based subjects to wear a Verily Study Watch for 23 h per day for up to two years with the actual average wear time being about 18 h. Derived measures were provided by Verily (Table 1) and accessed in November 2022. The derived data including 1-h interval timeseries data on physical activity (step count, walking minutes), sleep (total time, REM time, non-rapid eye movement (NREM) time, deep NREM time, light NREM time, wake after sleep onset (WASO), awakenings, sleep efficiency), and vital signs (pulse rate, mean root mean squared successive differences (RMSSD) (heart beat), median RMSSD, RMSSD variance) were available for 149 individuals diagnosed with PD, 158 individuals in the at-risk group, and 35 individuals without a diagnosis, covering a mean of 485 days. Six participants originally assigned to the at-risk group received a diagnosis of PD after recruitment but before digital data collection; these individuals were excluded from analysis. Not all 14 time series were available for every participant.

### Clinical and biological data

Data was downloaded from PPMI in 2021 and access to sequestered data was provided in 2023. The following clinical assessments were retrieved: University of Pennsylvania Smell Identification Test (UPSIT),^18^ Unified Parkinson Disease Rating Scale (UPDRS) scores,^19^ Scales for Outcomes in Parkinson's disease (SCOPA) autonome, and REM sleep behaviour disorder screening questionnaire (RBDSQ).^20^

The most recent minimum putamen striatal binding ratio (SBR) was calculated from DaTscan data and the binary indicator of DaTscan positivity was obtained from PPMI. These measurements were on average 0.33 ± 1.91 years before the digital data collection ended.

α-Synuclein SAA data from baseline CSF samples included the mean Fmax values across the three repetitions and the provided SAA classification. These measurements were on average 3.4 ± 1.38 years before the digital data collection ended. We used the most recent SAA data available at the time of this study; however, PPMI currently provides SAA results only from baseline samples. Future availability of more recent longitudinal data would allow further refinement of our analyses.

### Prodromal markers and risk factors

We retrieved all data necessary to calculate the MDS prodromal risk score^3^ (Table 3). We were not able to retrieve information on substantia nigra hyperechogenicity or urate levels, as these measurements were not uniformly collected in the PPMI study.

**Table 3:** Risk factors and prodromal markers.

Risk factors	
Age	Age at data retrieval date: 01.10.2021
Sex	Male
Pesticide exposure	FOUND questionnaire whether occupational exposure
Non-use of caffeine	FOUND questionnaire less than 6 cups of tea or 3 cups of coffee weekly
Never smoke	FOUND questionnaire not ever smoked regularly
Previous smoke	FOUND questionnaire ever smoked regularly and not smoke currently
Current smoke	FOUND current regular smoker
Physical inactivity	
1st degree relative with PD	Mother, father, or sibling with PD diagnosis (only used when PRS unavailable)
PRS	Polygenic risk score calculated with Nalls, et al.^21^
	Low if in lowest quartile, high if in highest quartile
Diabetes mellitus type II	Medical condition log searched for ‘(?!.∗pre)(?!.∗borderline)((.∗(II|2|two).∗Diabet.∗)|.∗Diabet.∗type.∗(II|2|two).∗)’
Prodromal markers	
Poven RBD	Medical condition log searched for ‘.∗(REM behavi|RBD|Rapid Eye).∗’ or listed under confirmed RBD in analytic dataset
RBD test	Ever scored higher than 5 on RBDSQ
Positive DaTscan	Visual inspection of DaTscan abnormal or minimum putamen SBR 2 std away from healthy control mean
Subthreshold parkinsonism	Ever UPDRS III score excluding postural and kinetic tremor above 6
Olfactory loss	Medical condition log searched for ‘.∗(hyposmia|anosmia).∗’ or listed under confirmed hyposmia in analytic dataset or ever scored below 1.5 std from age and sex matched mean^22^
Constipation	Medical condition log searched for ‘.∗constipation.∗’ OR UPDRS I 1.11 > 1
Excessive daytime sleepiness	Medical condition log searched for ‘.∗sleepiness.∗’ OR UPDRS I 1.13 > 1
Urinary dysfunction	Medical condition log searched for ‘(?!fecal).∗incontinence.∗’ OR UPDRS I 1.10 > 1
Orthostatic hypotension	Medical condition log searched for ‘.∗hypotension.∗’ OR UPDRS I 1.12 > 1
Erectile dysfunction	Medical condition log searched for ‘.∗erectile.∗’ OR SCOPA autonome 22 > 1
Depression	Medical condition log searched for ‘.∗(anxiety|depression).∗’ OR UPDRS I 1.3 > 1
Cognitive deficit	Ever cognitive categorisation listed as mild impairment or dementia

We describe the process of obtaining risk and prodromal markers from PPMI data. The selection of markers was taken from Heinzel, Berg.^3^

### Statistics

All analyses were performed in python 3.9 using sklearn 1.2.1^23^ for model training and evaluation, tsfresh 0.20.0^24^ for timeseries feature extraction, scipy 1.10.0 and pingouin 0.5.3 for statistical testing,^25^ and matplotlib 3.6.3 and seaborn 0.12.2 for creating figures. Data loading and manipulation has been facilitated through an adapted version of pypmi (https://github.com/aschalkamp/pypmi). All associated code will be made available at https://github.com/aschalkamp/PPMI_DigitalPaper, which can be used to replicate the performed analyses and retrieve the digital risk score. Analysis and reporting followed the TRIPOD+AI guidelines.

### Digital timeseries feature extraction

First, the overall mean over time was computed for each subject for each digital marker. The group of participants diagnosed with PD (68.48 ± 6.97 years) was significantly older than the at-risk group (64.49 ± 6.97) (Cohen's d = 0.51, p-value = 1.4 × 10^−5^) and had a higher proportion of males (0.6) than the at-risk group (0.37) (Table 2) thus linear models were fit on the healthy controls (N = 34/35) to identify the effect of age and sex on each marker. The resulting residuals were compared with two-sided t-tests with significant results defined as passing 0.05 FDR correction.

Second, tsfresh was applied for each subject for each raw, unadjusted digital feature to extract timeseries features. This resulted in 783 features such as maximum, minimum, skewness, kurtosis, and trend for each of the 14 digital timeseries, leading to a total of 10,962 features per subject.

### Risk models

The model described in the MDS research criteria^3^ was implemented using their suggested 80% probability threshold to binarise the resulting risk score. We include two versions of this: 1) a full version, 2) a restricted version excluding DaTscan positivity. The latter serves as a better representation when applied in the general population.

We computed the digital risk score by training elastic net logistic regression models identifying participants diagnosed with PD (N = 134) from healthy controls (N = 34) based on the computed digital timeseries features including only subjects with complete data (removed subjects PD = 15 and HC = 1). The at-risk group was not used for model training or validation at any point. A nested cross-validation was used with an inner and outer five-fold stratified split so internal validation was applied. For each fold in the outer cross-validation loop, the respective training dataset was used to standardise the data. The inner split was used to run a grid search to identify the best penalty parameter between 10^1^ and 10^4^ for alpha and between 0 and 1 for the L1 ratio (Table 4). The area under the precision-recall curve (AUPRC) was used as the evaluation score for model selection. We compared the model's performance to a baseline model using only age and male sex as predictors. The model's coefficients were assessed for stability and significance across folds after Bonferroni correction. The predicted probabilities were retrieved for all subjects, including the unseen evaluation set of at-risk subjects, as the average over the outer folds. The optimal threshold for identifying participants diagnosed with PD from healthy controls in terms of F1-score was found to be 0.54.

**Table 4:** Hyperparameters for machine learning models in gridsearch.

	Logistic regression	Polynomial support vector machine	RBF support vector machine	Random forest
Penalty	Elastic net			
C	np.logspace(1, 4, 5)	np.logspace(1, 4, 5)	np.logspace(1, 4, 5)	
L1–L2 ratio	np.linspace(0, 1, 5)			
Number of estimators				[50, 125, 200]
Maximum depth				[15, 57, 100]
Degree		[3, 4, 5]		

For each machine learning model, the hyperparameters are listed on which gridsearch was performed to identify the optimal values.

We performed additional analyses on the effect of the considered timeframe, the considered feature sets, and the applied machine learning model on the performance. All models were trained as outlined in the main manuscript in nested 5-fold cross validation where the inner loop performed gridsearch to identify the best hyperparameters. Performance was compared with area under the precision-recall curve (AUPRC) across the five outer test folds. First, we explored the performance of various machine learning models to identify participants diagnosed with PD from healthy controls. We compared logistic regression with elastic net penalty to random forests, support vector machines with polynomial kernel, and support vector machines with radial basis functions (Table 4). We chose logistic regression over the other models as it showed similar performance while being the simplest and most interpretable one. We explored how restriction to specific feature sets affects performance. We trained three models: one restricted to physical activity features, one to vital signs, and one to sleep. We further analysed how the digital risk score would perform if restricted to one week of data as compared to the model using the whole observation time of 1.3 years. For this, we identified the last hour when data was recorded for each subject and extracted the data up to seven days before. We then applied tsfresh as before, obtaining 783 features per timeseries and fitted the logistic regression model just as before.

### Comparison of predicted risks

109 of the 151 subjects in the at-risk group, who did not yet get a diagnosis of PD and for whom future phenoconversion status is unknown, had complete data available (Table 5). The digital risk score could be computed for 139 (incomplete data for 12) with an additional 30 being removed due to missing SAA, thus leading to the 109 subjects considered. We compared each pair of risk scores, pathological and biological markers with Pearson's correlation. Significant correlations are reported when passing 0.05 FDR correction. We assessed which known prodromal markers and risk factors (Table 3) were associated with higher digital risk scores using Welch's two-sided t-tests with 0.05 FDR correction. We only included the 14 markers for which 10 or more cases and controls were available. We further compared the estimated digital and MDS-based risk scores across biologically defined subgroups using two recently proposed frameworks for PD classification: the NSD staging system,^11^ which defines disease progression based on genetic risk (G), synuclein pathology (S), dopaminergic dysfunction (D), and clinical signs (C)^11^ and the biological SynNeurGe classification system^12^ using Welch's two-sided t-tests with 0.05 FDR correction. Following the SynNeurGe biological definition of PD, which combines genetics (G), α-synucleinopathy (S), and neurodegeneration (N), our at-risk cohort had 92 genetically predisposed individuals (G_P_+S−N−), 9 genetic Parkinson's type synucleinopathy (G_P_+S+N−), 3 Non-PD neurodegeneration (G_P_+S−N+), 4 sporadic PD (G_P_−S+N+), and 1 sporadic Parkinson's type synucleinopathy (G_P_−S+N−). Following the NSD staging system, 95 individuals are not assigned to any stage, 2 to 1A (S+N−C−), 8 to 2A (S+N−C+), and 4 to 2B (S+N+C+). This system does not allow for S− in presence of D+, which occurs in 4 individuals in our cohort, potentially due to the SAA being conducted at an earlier time point.

**Table 5:** Evaluation cohort.

	Proportion/mean	Std
**Demographics**		
Male	0.37	
Age [years]	64.62	6.86
**Cohort criteria**		
LRRK2	0.35	
GBA	0.61	
RBD PSG-proven	0.00	
Hyposmia	0.20	
**Prodromal markers**		
RBDSQ > 5	0.44	
Constipation	0.23	
Depression anxiety	0.37	
Excessive daytime sleepiness	0.24	
UPDRS > 6	0.28	
Erectile dysfunction	0.14	
Urinary dysfunction	0.17	
Orthostatic hypotension	0.16	
Diabetes II	0.05	
Cognitive impairment	0.20	
**Biological/pathological markers**		
SAA+	0.13	
DaT+	0.06	

The at-risk group on which the digital risk score is evaluated is presented with proportion of prodromal markers present, mean age, and sex information.

### Evaluation of risk scores

Assuming that DaTscan or CSF SAA serve as the gold standard for future conversion to PD, we assessed the performance of the digital risk model, the MDS prodromal model,^3^ and hyposmia in this scenario, computing recall, precision, and F1 score. We further assessed how a chaining of tests (i.e., first performing a digital screening and then sending all predicted positives to further tests) affected these performance metrics.

### Assessment of individuals with biological or pathological markers and low digital risk

We investigated individuals not identified by the digital risk model but that had positive CSF SAA (N = 4) or positive DaTscan (N = 4) in more detail. We computed Welch's two-sided t-test for the maximum ever recorded UPDRS III score comparing these individuals with individuals showing correctly identified (high digital risk and (DaTscan positive or SAA positive)). We repeated this analysis for hyposmia, and the restricted MDS prodromal risk score and report results as significant when passing 0.05 Bonferroni correction.

### Role of funders

The funders of the study had no role in study design, data collection, data analysis, data interpretation, or writing of the report.

### Ethics

All 48 participating PPMI sites received approval from their respective institutional review boards (IRBs), and written informed consent was obtained from all participants, including those enrolled in the at-risk cohort. The PPMI study is registered at ClinicalTrials.gov (NCT01141023). This analysis additionally used DaTscan and CSF α-synuclein SAA results from at-risk group, obtained from the PPMI database after approval by the PPMI Data Access Committee.

## Results

### Digital outcome measures capture differences in at-risk groups

The PPMI dataset provided a mean of 485 days of at home monitoring for 14 features describing physical activity, sleep, and vital signs in 1-h intervals (Table 1) for 343 subjects derived from the multi-sensor Verily Study Watch. The cohort included individuals diagnosed with PD (N = 149), the at-risk group (N = 158) which consists of individuals identified based on specific genetic (*GBA, LRRK2, SNCA*) and/or prodromal (polysomnography-proven RBD, hyposmia) markers, and unaffected controls (N = 35) (Tables 2 and 6). Note that not all 14 derived digital measures were available for every subject. Seven individuals of the at-risk group converted before digital data collection and were removed.

**Table 6:** Demographic characteristics.

Variable	Sex	Sample size	Age (Mean ± Std)	LRRK2 (%)	GBA (%)	RBD PSG-proven (%)	Hyposmia (%)	DaT+ (%)	SAA+ (%)
Healthy control	Male	19	66.16 ± 11.47	/	/	/	/	/	/
Healthy control	Female	16	67.31 ± 13.42	/	/	/	/	/	/
PD	Male	89	68.43 ± 8.81	/	/	/	/	/	/
PD	Female	60	68.56 ± 8.40	/	/	/	/	/	/
At-risk	Male	56	65.08 ± 7.54	59 (39.07%)	90 (59.6%)	2 (1.32%)	29 (19.21%)	14 (9.27%)	19 (12.58%)
At-risk	Female	95	64.14 ± 6.62	/	/	/	/	/	/
At-risk evaluation cohort (all)	Male	40	65.08 ± 7.54	38 (34.86%)	66 (60.55%)	0 (0%)	22 (20.18%)	7 (6.42%)	14 (12.84%)
At-risk evaluation cohort (all)	Female	69	64.14 ± 6.62	/	/	0 (0%)	/	/	/
At-risk evaluation cohort (LRRK2)	Male	13	6580 ± 8.07	13 (100%)	0 (0%)	0 (0%)	2 (15.38%)	0 (0%)	2 (15.38%)
At-risk evaluation cohort (LRRK2)	Female	25	64.19 ± 5.57	25 (100%)	0 (0%)	0 (0%)	4 (16%)	1 (8%)	2 (8%)
At-risk evaluation cohort (GBA)	Male	42	64.45 ± 7.27	0 (0%)	42 (100%)	0 (0%)	4 (16.67%)	0 (0%)	1 (4.17%)
At-risk evaluation cohort (GBA)	Female	24	63.82 ± 7.07	0 (0%)	24 (100%)	0 (0%)	7 (16.67%)	1 (2.38%)	4 (9.52%)
At-risk evaluation cohort (hyposmia)	Male	9	71.30 ± 6.33	2 (22.22%)	4 (44.44%)	0 (0%)	9 (100%)	2 (22.22%)	5 (55.56%)
At-risk evaluation cohort (hyposmia)	Female	13	64.40 ± 5.66	4 (30.77%)	7 (53.85%)	0 (0%)	13 (100%)	2 (15.38%)	5 (38.46%)
At-risk evaluation cohort (positive DaTscan)	Male	2	72.74 ± 0.13	0 (0%)	0 (0%)	0 (0%)	2 (100%)	2 (100%)	2 (100%)
At-risk evaluation cohort (positive DaTscan)	Female	5	69.97 ± 4.15	2 (40%)	1 (20%)	0 (0%)	2 (40%)	5 (100%)	2 (40%)
At-risk evaluation cohort (positive SAA)	Male	6	71.22 ± 7.47	2 (25%)	4 (50%)	0 (0%)	5 (83.33%)	2 (25%)	6 (100%)
At-risk evaluation cohort (positive SAA)	Female	8	68.71 ± 5.28	2 (33.33%)	1 (16.67%)	0 (0%)	5 (62.5%)	2 (33.33%)	8 (100%)

For each cohort the sample size, proportion male and mean age at digital data collection is shown. This is for the overall cohort and then we show those also for the evaluation cohort which was selected from the at-risk cohort as those having all data available. We further show the percentages of LRRK2, GBA, hyposmia, DaT+, and SAA+ per cohort.

**Fig. 1:**
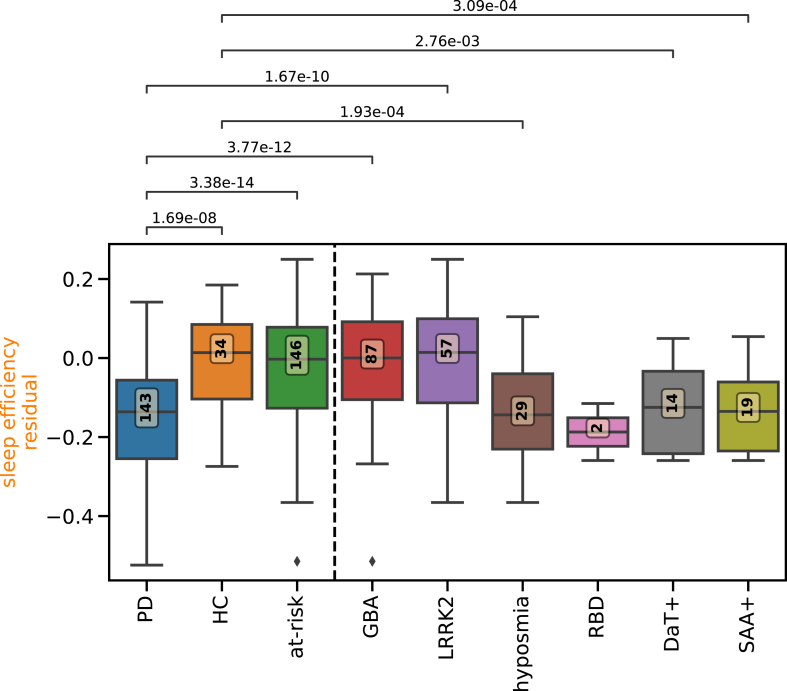
**Digital measures capture differences in at-risk groups.** The boxplots show the residual overall mean of digitally tracked sleep efficiency adjusted for age and sex with parameters learnt from a linear regression on the healthy controls. The overall mean is computed over the whole observation time per subject for each group. The boxplots depict the group median and quartiles per group with the whiskers showing the Q3 + 1.5 interquartile range (IQR) and Q1 − 1.5 IQR (Parkinson's disease cases: PD; healthy controls: HC; carriers of genetic risk alleles or prodromal symptoms without a diagnosis of PD: GBA, LRRK2, hyposmia, polysomnography-proven RBD, positive DaTscan, positive SAA; union of these: at-risk). The number in the yellow box indicates the number of individuals per group. Group differences were calculated with two-sided t-test comparing PD and HC to each of the at-risk groups. Lines and numbers show significant differences with 0.05 FDR corrected p-values.

We computed the mean for each digital measure over the complete period of observation. Comparing these average measures demonstrated that all physical activity measures were reduced in participants diagnosed with PD compared to controls (Fig. 1, Supplementary Table S1, Supplementary Figure S1): step count (Cohen's d = 1.24, p-value = 9.57 × 10^−10^, two-sided t-test), walking minutes (Cohen's d = 1.04, p-value = 2.03 × 10^−7^, two-sided t-test). Four of the eight sleep measures were also significantly lower in individuals diagnosed with PD compared to controls; sleep length (Cohen's d = 0.62, p-value = 2.56 × 10^−3^, two-sided t-test), sleep efficiency (Cohen's d = 1.15, p-value = 1.69 × 10^−8^, two-sided t-test), REM (Cohen's d = 1.2, p-value = 4.11 × 10^−9^, two-sided t-test), and deep NREM sleep length (Cohen's d = 0.94, p-value = 3.37 × 10^−6^, two-sided t-test). None of the four digital vital signs were significantly different between individuals with Parkinson's disease and healthy controls.

Due to the heterogeneity of the at-risk group, we split the group by specific identifiers. The whole at-risk group including genetic carriers and those with prodromal symptoms did not show any differences to healthy controls. Differences between controls and the at-risk subgroups were observed for sleep efficiency, which was significantly reduced in individuals with hyposmia (Cohen's d = 1.04, p-value = 1.93 × 10^−4^, two-sided t-test), positive DaTscan (Cohen's d = 1.05, p-value = 2.76 × 10^−3^, two-sided t-test), or positive SAA (Cohen's d = 1.14, p-value = 3.09 × 10^−4^, two-sided t-test) (Fig. 1). Individuals with hyposmia showed significant differences to the controls in five measures, individuals with SAA positivity showed differences in four, and individuals with DaTscan positivity differed in one measure (Supplementary Figure S1, Supplementary Table S1). Overall, the digital measures hold information relevant to PD and its prodromal markers.

### Long-term digital risk score identifies Parkinson's disease

**Fig. 2:**
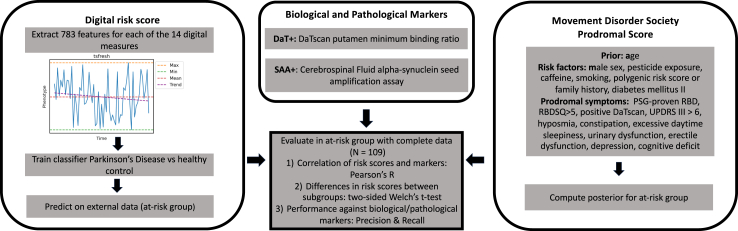
**Derivation of risk scores and overview of statistical analyses.** Overview of analysis. Derivation of risk scores and biological and pathological markers. Illustration of performed tests and modelling.

**Fig. 3:**
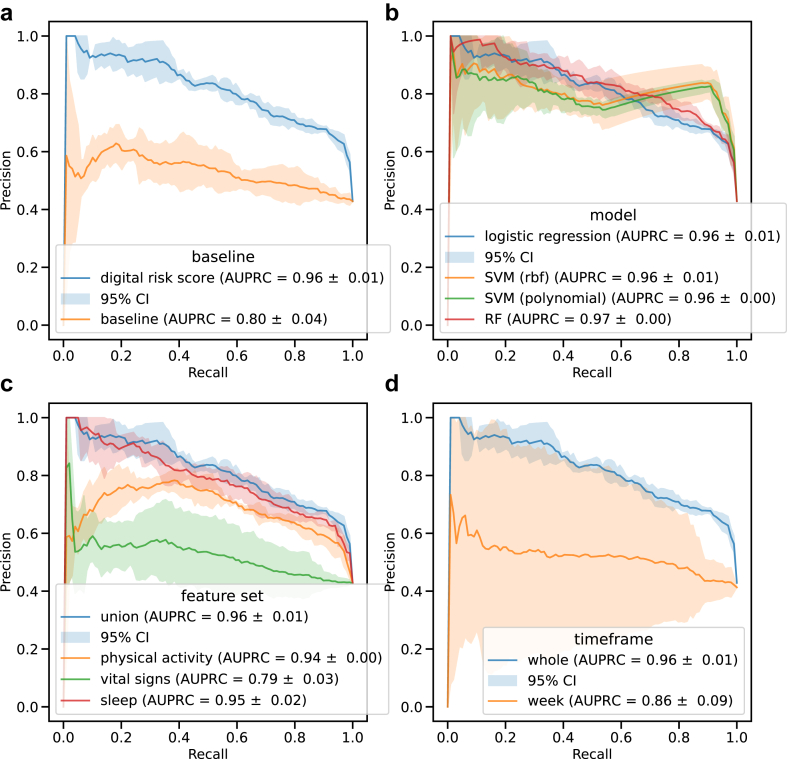
**Performance of digital risk models.** The performances for the digital risk score models are shown compared to a) baseline, b) other machine learning models, c) other feature sets, and d) other considered time frames. The precision-recall curves are shown as the mean on the outer 5-folds of the nested cross-validation. The shaded area displays the 95% Confidence Interval (CI). For each classifier, the legend shows the mean area under the precision-recall curve (AUPRC) with the standard deviation. SVM: support vector machine, rbf: radial basis function, RF: random forest.

We obtained digital risk scores (Fig. 2) from models trained on the 783 timeseries features as extracted with tsfresh for each of the 14 digital measures (Supplementary Table S2). The logistic regression model was trained to identify participants diagnosed with PD (N = 135) from healthy controls (N = 34), leaving the at-risk group (N = 109) as a separate evaluation dataset not seen during training or validation (Table 6). The digital risk model (AUPRC = 0.96 ± 0.01) significantly outperformed the baseline model (AUPRC = 0.8 ± 0.04, p-value = 8 × 10^−6^) (Fig. 3a, Supplementary Figure S2a). Consistently selected features predominantly originated from REM sleep time (41.39%) and step count (48.28%) (Supplementary Figure S3). Logistic regression performed on-par with other machine learning methods (Fig. 3a, Supplementary Figure S2a). Ablation analyses on the considered feature sets revealed the union of all features to perform better than models trained on subsets (Fig. 3c, Supplementary Figure S2c) with a model only trained on vital signs performing worst. Additional analyses on the considered timeframe showed longer timespans to better distinguish between participants diagnosed with PD and control (Fig. 3d, Supplementary Figure S2d).

### Digital risk score relates to Movement Disorder Society research criteria

**Fig. 4:**
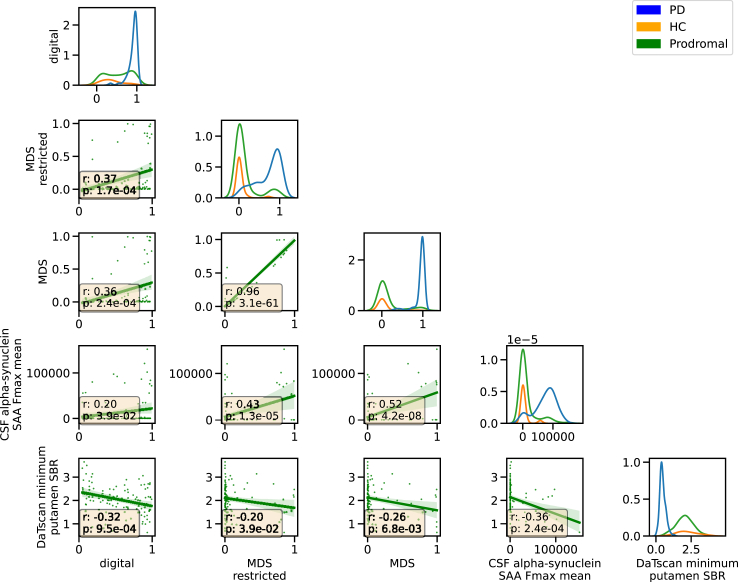
**Digital risk score correlates with MDS prodromal score and biological markers.** The relation between the different risk scores and biological markers is shown. On the diagonal, the distribution for each diagnostic group is displayed (PD: diagnosed Parkinson's disease, HC: healthy control, Prodromal: at-risk cohort of genetic mutations carriers and individuals with prodromal symptoms). The scatterplot shows the relation between each pair of markers (digital: digital risk score, MDS restricted: Movement Disorder Society (MDS) prodromal risk score without DaTscan information, MDS: MDS prodromal risk score with DaTscan information if available, CSF α-synuclein SAA Fmax mean: mean value of the five repetitions of seed amplification assay (SAA) on CSF, DaTscan minimum putamen: minimum of hemispheres dopaminergic imaging scan (DaTscan) striatal binding ratio (SBR^10^) in putamen) in the at-risk group (N = 109) with the text box displaying the Pearson r coefficient and the 0.05 FDR corrected p-value.

We computed a prodromal risk score using the model defined in the MDS research criteria^3^ (Fig. 2, Table 3). In our at-risk evaluation cohort (Table 5), our digital risk score was significantly correlated with the MDS risk score (Pearson's r = 0.36, p-value = 2.43 × 10^−4^, N = 109, t-test) and the restricted MDS score excluding DaTscan information (Pearson's r = 0.37, p-value = 1.65 × 10^−4^, t-test, Fig. 4, Supplementary Table S3).

**Fig. 5:**
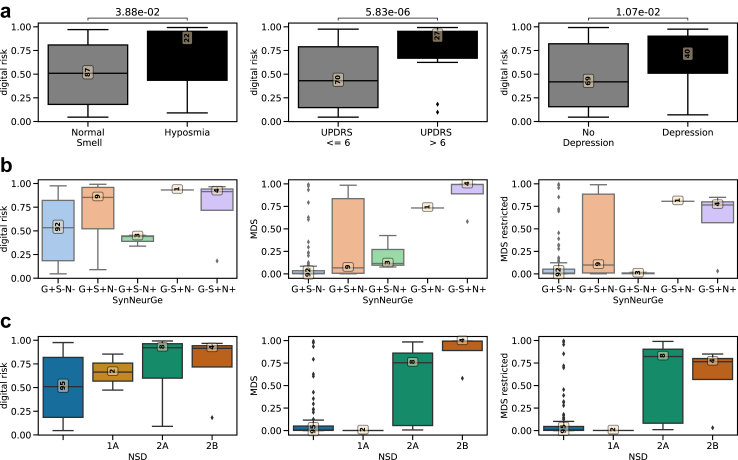
**Digital risk score is increased in individuals with known prodromal markers and biological classification groups.** a) The boxplots show the difference in digital risk score between carriers and non-carriers (x-axis). The 0.05 FDR-corrected p-value from two-sided Welch t-test is shown. The yellow box presents the number of subjects in each group. This plot shows those prodromal markers and risk factors from the model included in Heinzel, Berg^3^ that were significant after FDR-correction. A complete table with statistical results can be found in Supplementary Table S4. b) The distribution of risk scores for the different biological groups defined by SynNeurGe^12^ for the digital, the MDS, and the restricted MDS risk scores. c) The distribution of the risk score for the different biological stages defined by NSD^11^ for the digital, MDS, and the restricted MDS risk scores.

We investigated which known risk factors and prodromal symptoms implied significant differences in digital risk. Individuals with subthreshold Parkinsonism (UPDRS III > 6) (Cohen's d = 1.11, p-value = 5.83 × 10^−6^, Welch's t-test), hyposmia (Cohen's d = 0.67, p-value = 3.88 × 10^−2^, Welch's t-test), or depression (Cohen's d = 0.62, p-value = 1.07 × 10^−2^, Welch's t-test) had higher digital risk scores (Fig. 5a, Supplementary Tables S4 and S5).

### Digital risk score represents neurodegeneration and synucleinopathy

The digital risk score was correlated weakly but significantly with the minimum putamen SBR derived from DaTscan (Pearson's r = −0.32, p-value = 9.49 × 10^−4^, t-test) and the CSF α-synuclein SAA (Pearson's r = 0.2, p-value = 3.9 × 10^−2^, t-test, Fig. 4, Supplementary Table S3). Compared to the restricted MDS risk score, which also correlated to a similar degree with these biological and pathological markers, the digital risk score showed a stronger correlation with DaTscan (−0.32 vs −0.2) but a weaker one with CSF α-synuclein SAA (0.2 vs 0.43).

Recent efforts have been made to derive biological definitions for PD, we investigated the digital risk score against two systems: SynNeurGe^12^ and NSD.^11^ Following the SynNeurGe definitions, our digital risk score was highest in participants with sporadic PD (0.74 ± 0.38 (N = 4)) and lowest for groups without synucleinopathy (G_P_+S−N−: 0.52 ± 0.32 (N = 92), G_P_+S−N+: 0.41 ± 0.06 (N = 3)) (Fig. 5b). Similar observations can be made for the MDS criteria. Following the NDS staging system, the digital risk score increases with each stage whereas the MDS prodromal score shows a steep increase only from stage 2 onwards (Fig. 5c).

### Digital risk score more sensitively detects synucleinopathy and neurodegeneration than MDS criteria

As no individuals in our at-risk group received a diagnosis of PD after digital data collection, we had no gold standard information on phenoconversion available. Instead, we assessed the risk models for their ability to identify synucleinopathy and neurodegeneration as measured with α-synuclein SAA and DaTscan, which are known to already be altered in the prodromal stage.^9^^,^^10^^,^^26^

The digital model identified 28.57% more of the individuals with synucleinopathy or neurodegeneration than the MDS model (Table 7, Supplementary Table S6). While the digital risk score showed higher recall than the MDS model, it had lower precision (Table 7, Supplementary Table S6). Compared to hyposmia, as determined by UPSIT test and medical records (Table 3), the digital model had equal recall, except for DaTscan positivity where hyposmia identified one additional participant correctly (Supplementary Table S6). Generally, hyposmia performed better or equal to the digital risk score. The two tests, however, identified distinct individuals for SAA positivity with a combined risk identifying 12 of the 14 SAA positive cases increasing the recall by 0.12.

**Table 7:** The digital risk score sensitively identifies people with biological or pathological markers of Parkinson's disease.

	TN	FP	FN	TP	Precision	Recall	F1 score
MDS	89	3	11	6	0.67	0.35	0.46
MDS restricted	88	4	11	6	0.6	0.35	0.44
Hyposmia	80	12	7	10	0.45	0.59	0.51
Digital	46	46	7	10	0.18	0.59	0.27
Digital + hyposmia	42	50	5	12	0.19	0.71	0.3
SAA	92	0	3	14	1	0.82	0.9
DaTscan	92	0	10	7	1	0.41	0.58

Due to a lack of future conversion information, we display the performance of each risk score against the combination of DaTscan positivity and SAA positivity. The number of true positives (TP), false positives (FP), true negatives (TN), and false negatives (FN) is shown alongside precision, recall, and F1 score. Importantly, MDS includes information on DaTscan positivity in its model, hence, we included a restricted version without this information as well.

The risk models could be biased towards identifying only those individuals already presenting with minor motor impairments (Supplementary Table S7). The individuals that the digital model did not identify but that had either positive SAA or DaT, had lower maximum UPDRS III scores (mean = 1.86 ± 2.27, N = 7) than the ones correctly identified by the digital risk (mean = 15.7 ± 11.33, N = 10) (Cohen's d = 1.56, p-value = 3.74 × 10^−3^, Welch's t-test). Hyposmia (Cohen's d = 1.65, p-value = 2.58 × 10^−3^, Welch's t-test) and the restricted MDS prodromal risk score (Cohen's d = 3.1, p-value = 2.15 × 10^−4^, Welch's t-test) showed this same bias towards individuals with higher UPDRS III scores being identified and those with lower being missed.

Of the 109 at-risk subjects the digital risk score identified 51.38% (N = 56) as high risk whereas the MDS research criteria only flagged 8.26% (N = 9), which can also be seen in the bi-modal distribution for the digital risk score compared to a heavy-tailed one for the MDS model (Fig. 4). Sequential testing could be applied such that all predicted positive cases from the digital risk model would be sent for further testing with hyposmia, with the final examination being performed with CSF α-synuclein SAA or DaTscan. Favouring the sensitive digital risk over the MDS score for such a sequential screening is indicated by 11 more individuals with either DaT+ or SAA+ being identified rather than missed with the MDS (Supplementary Figure S4).

## Discussion

We leveraged the 1.3-years of continuously collected smartwatch data from the PPMI cohort to derive a digital risk score. Individuals with known prodromal markers, subthreshold Parkinsonism or hyposmia, demonstrated an increased digital risk. The digital risk score more sensitively detected neurodegeneration or synucleinopathy than the MDS research criteria.^3^

We have previously demonstrated the ability to identify those who will go on to receive a future diagnosis of PD using one week of accelerometer data.^13^ Here, we evaluated long-term digital markers derived from a multi-sensor device worn by individuals harbouring genetic risk variants or prodromal markers for PD. We assessed the improvement in using the whole observation time versus the last week of data available and found a significant improvement for the long-term risk. The addition of the PPG sensor allowed the extraction of sleep stages and vital signs, which significantly contributed to the digital risk (Fig. 3, Supplementary Figures S2 and S3). The extended timeframe and measures thus contributed to an improved digital risk score.

With the digital risk correlating with biological and pathological markers of PD, its relevance for early screening was further highlighted. Compared to the MDS research criteria, the digital risk score had a higher recall for CSF α-synuclein SAA positivity (increase by 0.29) and DaTscan positivity (increase by 0.29). The MDS score poses much importance on the RBD status does not commonly occur in LRRK2 carriers with a PD diagnosis.^27^ Previous research reported hyposmia as a good predictor for DaTscan positivity^28^ with a recall of 0.96 and a precision of 0.14. In our dataset, hyposmia achieved a recall of 0.57 and a precision of 0.18. This discrepancy could be attributed to a different method for ascertainment of hyposmia or population characteristics. Generally, hyposmia performed as well or better than the digital risk score. However, we noted that distinct individuals were identified for CSF α-synuclein SAA positivity by the digital risk score and hyposmia, with a combination identifying 85.71% of CSF α-synuclein SAA positive cases.

The digital risk score identified half of the at-risk group as high-risk. This could indicate a high rate of false positives, placing a burden on healthcare systems to undertake additional screening tests, and anxiety for the individuals incorrectly identified as being at high-risk. Prior to additional invasive testing being undertaken based on digital risk, further clinical examination should be performed (including testing for hyposmia), aligning with current recruiting strategies for prodromal cohorts.^29^ The true rate of false positives remains to be determined due to the missing information on future phenoconversion in the current dataset. Although CSF α-synuclein SAA and DaTscan are good markers for the neuropathological changes associated with PD, they are not diagnostic tools.^10^ For example, LRRK2 carriers who have a diagnosis of PD do not necessarily have positive SAA with around 33% testing negative.^10^ Ongoing follow-up is thus needed to assess the true predictive performance of the risk scores based on phenoconversion.

A digital risk score could be integrated in a sequential screening pipeline as the first test to be performed. Due to the passive data collection and ongoing recording of data, a digital risk score can easily be calculated on a rolling basis. This is in contrast to the MDS criteria that include various clinical tests to be carried out, including DaTscan, which is generally only performed when PD is already suspected.^30^ The sequential screening pipeline could incorporate DaTscan as a secondary test, which has previously been proposed.^31^ The first test in this pipeline should be accessible, cheap, and scalable. Hyposmia is currently the most promising early screening marker for PD with the test being low-cost and easily accessible.^28^^,^^32^ Here, we showed that a digital risk score could offer an alternative or addition to this test with passively collected digital data having the advantage of continuous and passive longer-term follow-up. As our analysis showed that the union of hyposmia and the digital risk identified SAA or DaTscan positivity the best, a combination of these two would increase their individual recall from 0.59 each to 0.71 when combined while remaining cost efficient.

The primary limitations of this study relate to data availability and choice of methodology. Due to the Verily Study Watch only being introduced 10 years after the start of the PPMI study, for some individuals the different data modalities have been collected several years apart, limiting the comparability between modalities. With the digital risk being collected most recently, its detection performance could be attributed to individuals being potentially closer to phenoconversion. The PPMI at-risk group currently only includes 28 individuals known to have subsequently received a diagnosis (converters) of which only seven had digital data available with six converting prior to data collection and one without a known conversion date, limiting the assessment of the true risk of developing PD. Notable sample size restrictions limit the power and generalisability of our results. The control group available for model training and validation was restricted to 34 healthy controls due to only few healthy controls wearing smartwatches within the PPMI study, restricting our analysis and prohibiting age-sex matching. The evaluation cohort of 109 subjects only included 7 DaT+ and 14 SAA+ cases. Our study was further limited to the derived features provided by Verily, the code for which is proprietary, limiting reproducibility in other cohorts. As sleep scores differ highly between devices and employed processing and have not reached the same performance as achieved with PSG,^33^ the included sleep features should be interpreted with this variability in mind. Further potential confounders that have not been assessed include medication and comorbidities. Race and ethnicity have not been assessed in this analysis and the representativeness of the reported results remains to be assessed. Finally, due to lack of an independent validation cohort from a separate study, our findings remain to be replicated in the future.

In conclusion, long-term digital monitoring can inform disease risk outperforming the MDS research criteria for prodromal PD to detect neurodegeneration or synucleinopathy. Sequential screening methods should be further developed and implemented to facilitate recruitment of individuals into future clinical trials focussed on dopaminergic deficits or α-synucleinopathy.

## Contributors

A-K.S. and C.S. participated in designing the study, topic definition, and review of relevant studies. Machine learning models and statistical analyses were designed and implemented by A-K.S. Figures and tables were done by A-K.S. with the support of C.S. A-K.S. wrote the first draft. A-K.S., C.S., N.A.H., K.J.P., V.E-P., and P.B. contributed to subsequent versions of the manuscript. All authors read and approved the final version of the manuscript, all authors have a clear understanding of the content, results, and conclusions of the study and agree to submit this manuscript for publication. The corresponding author (C.S.) declares that all authors listed meet the authorship criteria and that no other authors involved in this study are omitted. C.S. is ultimately responsible for this article.

## Data sharing statement

This analysis used DaTscan and α-synuclein SAA results for at-risk participants, obtained from PPMI upon request after approval by the PPMI Data Access Committee. Data used in the preparation of this article were obtained in November 2022 from the Parkinson's Progression Markers Initiative (PPMI) database (www.ppmi-info.org/access-data-specimens/download-data), RRID: SCR_006431. Sequestered data was given access to October 2023. For up-to-date information on the study, visit www.ppmi-info.org. All associated code to reproduce the analyses performed here will be made publicly available upon publication (https://github.com/aschalkamp/DigitalPPMI).

## Declaration of interests

All authors declare no competing interests.
