# Supplementary material for: Wearables-derived risk score for unintrusive detection of α-synuclein aggregation or dopaminergic deficit
Source: eBioMedicine. 2025 Jun 6;117:105782. doi: 10.1016/j.ebiom.2025.105782 (PMC12205697; doi:10.1016/j.ebiom.2025.105782)
Supplement: Supplementary Tables and Figures [file mmc1.docx]

**Supplemental Material**

Table of Contents

[Supplemental Tables 1](#_Toc197000756)

[**Supplementary Table 1: Significant differences in digital markers between groups** 2](#_Toc197000757)

[**Supplementary Table 2: Extracted digital timeseries features** 2](#_Toc197000758)

[**Supplementary Table 3: Correlation of risk scores and biological measures** 2](#_Toc197000759)

[**Supplementary Table 4: Differences in digital score between risk factor and prodromal symptom carriers** 3](#_Toc197000760)

[**Supplementary Table 5: Mean risk score for each at-risk group** 3](#_Toc197000761)

[**Supplementary Table 6: Performance of the risk scores/markers in identifying DaTscan and alpha-synuclein positivity** 4](#_Toc197000762)

[**Supplementary Table 7: Differences in UPDRS III between individuals at biological and pathological risk identified and missed by digital risk score** 5](#_Toc197000763)

[Supplemental Figures 6](#_Toc197000764)

[**Supplementary Figure 1: Mean digital markers are affected in people diagnosed with PD** 6](#_Toc197000765)

[**Supplementary Figure 2: Performance of digital risk models in terms of area under receiver operator curve** 7](#_Toc197000766)

[**Supplementary Figure 3: Significant predictors of digital risk model** 8](#_Toc197000767)

[**Supplementary Figure 4: Digital risk score as a sensitive screening tool in a sequence of testing** 9](#_Toc197000768)

[References 9](#_Toc197000769)

# Supplemental Tables

|  | **proportion/mean** | **std** |
| --- | --- | --- |
| **Demographics** | |  |
| male | 0.366972 |  |
| age [ years] | 64.61577 | 6.86065 |
| **Cohort criteria** | |  |
| LRRK2 | 0.348624 |  |
| GBA | 0.605505 |  |
| RBD PSG-proven | 0 |  |
| hyposmia | 0.201835 |  |
| **prodromal markers** | |  |
| RBDSQ>5 | 0.444444 |  |
| constipation | 0.229358 |  |
| DepressionAnxiety | 0.366972 |  |
| ExcessiveDaytimeSleepiness | 0.238532 |  |
| UPDRS>6 | 0.278351 |  |
| ErectileDysfunction | 0.137615 |  |
| UrinaryDysfunction | 0.174312 |  |
| OrthostaticHypotension | 0.155963 |  |
| DiabetesII | 0.04878 |  |
| cognitive impairment | 0.201835 |  |
| **Biological/pathological markers** | | |
| SAA+ | 0.12844 |  |
| DaT+ | 0.06422 |  |

## **Supplementary Table 1: Significant differences in digital markers between groups**

The mean value per residual mean digital marker corrected for age and sex is shown for the healthy controls, the diagnosed PD, and the prodromal GBA, LRRK2, SNCA, hyposmia, RBD, and DaTscan positive cases together with the sample size per group. We show the statistics of the two sided T-test as the t-statistic and p-value. The digital markers are here the mean over the whole observation time for each individual.

|  | tsfresh feature | |
| --- | --- | --- |
| 0 | abs_energy |  |
| 1 | absolute_maximum | |
| 2 | absolute_sum_of_changes | |
| 3 | agg_autocorrelation__f_agg_"mean"__maxlag_40 | |
| 4 | agg_autocorrelation__f_agg_"median"__maxlag_40 | |
| 5 | agg_autocorrelation__f_agg_"var"__maxlag_40 | |
| 6 | agg_linear_trend__attr_"intercept"__chunk_len_10__f_agg_"max" | |
| 7 | agg_linear_trend__attr_"intercept"__chunk_len_10__f_agg_"mean" | |
| 8 | agg_linear_trend__attr_"intercept"__chunk_len_10__f_agg_"min" | |
| 9 | agg_linear_trend__attr_"intercept"__chunk_len_10__f_agg_"var" | |
| 10 | agg_linear_trend__attr_"intercept"__chunk_len_50__f_agg_"max" | |
| 11 | agg_linear_trend__attr_"intercept"__chunk_len_50__f_agg_"mean" | |
| 12 | agg_linear_trend__attr_"intercept"__chunk_len_50__f_agg_"min" | |
| 13 | agg_linear_trend__attr_"intercept"__chunk_len_50__f_agg_"var" | |
| 14 | agg_linear_trend__attr_"intercept"__chunk_len_5__f_agg_"max" | |
| 15 | agg_linear_trend__attr_"intercept"__chunk_len_5__f_agg_"mean" | |
| 16 | agg_linear_trend__attr_"intercept"__chunk_len_5__f_agg_"min" | |
| 17 | agg_linear_trend__attr_"intercept"__chunk_len_5__f_agg_"var" | |
| 18 | agg_linear_trend__attr_"rvalue"__chunk_len_10__f_agg_"max" | |
| 19 | agg_linear_trend__attr_"rvalue"__chunk_len_10__f_agg_"mean" | |
| 20 | agg_linear_trend__attr_"rvalue"__chunk_len_10__f_agg_"min" | |
| 21 | agg_linear_trend__attr_"rvalue"__chunk_len_10__f_agg_"var" | |
| 22 | agg_linear_trend__attr_"rvalue"__chunk_len_50__f_agg_"max" | |
| 23 | agg_linear_trend__attr_"rvalue"__chunk_len_50__f_agg_"mean" | |
| 24 | agg_linear_trend__attr_"rvalue"__chunk_len_50__f_agg_"min" | |
| 25 | agg_linear_trend__attr_"rvalue"__chunk_len_50__f_agg_"var" | |
| 26 | agg_linear_trend__attr_"rvalue"__chunk_len_5__f_agg_"max" | |
| 27 | agg_linear_trend__attr_"rvalue"__chunk_len_5__f_agg_"mean" | |
| 28 | agg_linear_trend__attr_"rvalue"__chunk_len_5__f_agg_"min" | |
| 29 | agg_linear_trend__attr_"rvalue"__chunk_len_5__f_agg_"var" | |
| 30 | agg_linear_trend__attr_"slope"__chunk_len_10__f_agg_"max" | |
| 31 | agg_linear_trend__attr_"slope"__chunk_len_10__f_agg_"mean" | |
| 32 | agg_linear_trend__attr_"slope"__chunk_len_10__f_agg_"min" | |
| 33 | agg_linear_trend__attr_"slope"__chunk_len_10__f_agg_"var" | |
| 34 | agg_linear_trend__attr_"slope"__chunk_len_50__f_agg_"max" | |
| 35 | agg_linear_trend__attr_"slope"__chunk_len_50__f_agg_"mean" | |
| 36 | agg_linear_trend__attr_"slope"__chunk_len_50__f_agg_"min" | |
| 37 | agg_linear_trend__attr_"slope"__chunk_len_50__f_agg_"var" | |
| 38 | agg_linear_trend__attr_"slope"__chunk_len_5__f_agg_"max" | |
| 39 | agg_linear_trend__attr_"slope"__chunk_len_5__f_agg_"mean" | |
| 40 | agg_linear_trend__attr_"slope"__chunk_len_5__f_agg_"min" | |
| 41 | agg_linear_trend__attr_"slope"__chunk_len_5__f_agg_"var" | |
| 42 | agg_linear_trend__attr_"stderr"__chunk_len_10__f_agg_"max" | |
| 43 | agg_linear_trend__attr_"stderr"__chunk_len_10__f_agg_"mean" | |
| 44 | agg_linear_trend__attr_"stderr"__chunk_len_10__f_agg_"min" | |
| 45 | agg_linear_trend__attr_"stderr"__chunk_len_10__f_agg_"var" | |
| 46 | agg_linear_trend__attr_"stderr"__chunk_len_50__f_agg_"max" | |
| 47 | agg_linear_trend__attr_"stderr"__chunk_len_50__f_agg_"mean" | |
| 48 | agg_linear_trend__attr_"stderr"__chunk_len_50__f_agg_"min" | |
| 49 | agg_linear_trend__attr_"stderr"__chunk_len_50__f_agg_"var" | |
| 50 | agg_linear_trend__attr_"stderr"__chunk_len_5__f_agg_"max" | |
| 51 | agg_linear_trend__attr_"stderr"__chunk_len_5__f_agg_"mean" | |
| 52 | agg_linear_trend__attr_"stderr"__chunk_len_5__f_agg_"min" | |
| 53 | agg_linear_trend__attr_"stderr"__chunk_len_5__f_agg_"var" | |
| 54 | approximate_entropy__m_2__r_0.1 | |
| 55 | approximate_entropy__m_2__r_0.3 | |
| 56 | approximate_entropy__m_2__r_0.5 | |
| 57 | approximate_entropy__m_2__r_0.7 | |
| 58 | approximate_entropy__m_2__r_0.9 | |
| 59 | ar_coefficient__coeff_0__k_10 | |
| 60 | ar_coefficient__coeff_10__k_10 | |
| 61 | ar_coefficient__coeff_1__k_10 | |
| 62 | ar_coefficient__coeff_2__k_10 | |
| 63 | ar_coefficient__coeff_3__k_10 | |
| 64 | ar_coefficient__coeff_4__k_10 | |
| 65 | ar_coefficient__coeff_5__k_10 | |
| 66 | ar_coefficient__coeff_6__k_10 | |
| 67 | ar_coefficient__coeff_7__k_10 | |
| 68 | ar_coefficient__coeff_8__k_10 | |
| 69 | ar_coefficient__coeff_9__k_10 | |
| 70 | augmented_dickey_fuller__attr_"pvalue"__autolag_"AIC" | |
| 71 | augmented_dickey_fuller__attr_"teststat"__autolag_"AIC" | |
| 72 | augmented_dickey_fuller__attr_"usedlag"__autolag_"AIC" | |
| 73 | autocorrelation__lag_0 | |
| 74 | autocorrelation__lag_1 | |
| 75 | autocorrelation__lag_2 | |
| 76 | autocorrelation__lag_3 | |
| 77 | autocorrelation__lag_4 | |
| 78 | autocorrelation__lag_5 | |
| 79 | autocorrelation__lag_6 | |
| 80 | autocorrelation__lag_7 | |
| 81 | autocorrelation__lag_8 | |
| 82 | autocorrelation__lag_9 | |
| 83 | benford_correlation | |
| 84 | binned_entropy__max_bins_10 | |
| 85 | c3__lag_1 |  |
| 86 | c3__lag_2 |  |
| 87 | c3__lag_3 |  |
| 88 | change_quantiles__f_agg_"mean"__isabs_False__qh_0.2__ql_0.0 | |
| 89 | change_quantiles__f_agg_"mean"__isabs_False__qh_0.4__ql_0.0 | |
| 90 | change_quantiles__f_agg_"mean"__isabs_False__qh_0.4__ql_0.2 | |
| 91 | change_quantiles__f_agg_"mean"__isabs_False__qh_0.6__ql_0.0 | |
| 92 | change_quantiles__f_agg_"mean"__isabs_False__qh_0.6__ql_0.2 | |
| 93 | change_quantiles__f_agg_"mean"__isabs_False__qh_0.6__ql_0.4 | |
| 94 | change_quantiles__f_agg_"mean"__isabs_False__qh_0.8__ql_0.0 | |
| 95 | change_quantiles__f_agg_"mean"__isabs_False__qh_0.8__ql_0.2 | |
| 96 | change_quantiles__f_agg_"mean"__isabs_False__qh_0.8__ql_0.4 | |
| 97 | change_quantiles__f_agg_"mean"__isabs_False__qh_0.8__ql_0.6 | |
| 98 | change_quantiles__f_agg_"mean"__isabs_False__qh_1.0__ql_0.0 | |
| 99 | change_quantiles__f_agg_"mean"__isabs_False__qh_1.0__ql_0.2 | |
| 100 | change_quantiles__f_agg_"mean"__isabs_False__qh_1.0__ql_0.4 | |
| 101 | change_quantiles__f_agg_"mean"__isabs_False__qh_1.0__ql_0.6 | |
| 102 | change_quantiles__f_agg_"mean"__isabs_False__qh_1.0__ql_0.8 | |
| 103 | change_quantiles__f_agg_"mean"__isabs_True__qh_0.2__ql_0.0 | |
| 104 | change_quantiles__f_agg_"mean"__isabs_True__qh_0.4__ql_0.0 | |
| 105 | change_quantiles__f_agg_"mean"__isabs_True__qh_0.4__ql_0.2 | |
| 106 | change_quantiles__f_agg_"mean"__isabs_True__qh_0.6__ql_0.0 | |
| 107 | change_quantiles__f_agg_"mean"__isabs_True__qh_0.6__ql_0.2 | |
| 108 | change_quantiles__f_agg_"mean"__isabs_True__qh_0.6__ql_0.4 | |
| 109 | change_quantiles__f_agg_"mean"__isabs_True__qh_0.8__ql_0.0 | |
| 110 | change_quantiles__f_agg_"mean"__isabs_True__qh_0.8__ql_0.2 | |
| 111 | change_quantiles__f_agg_"mean"__isabs_True__qh_0.8__ql_0.4 | |
| 112 | change_quantiles__f_agg_"mean"__isabs_True__qh_0.8__ql_0.6 | |
| 113 | change_quantiles__f_agg_"mean"__isabs_True__qh_1.0__ql_0.0 | |
| 114 | change_quantiles__f_agg_"mean"__isabs_True__qh_1.0__ql_0.2 | |
| 115 | change_quantiles__f_agg_"mean"__isabs_True__qh_1.0__ql_0.4 | |
| 116 | change_quantiles__f_agg_"mean"__isabs_True__qh_1.0__ql_0.6 | |
| 117 | change_quantiles__f_agg_"mean"__isabs_True__qh_1.0__ql_0.8 | |
| 118 | change_quantiles__f_agg_"var"__isabs_False__qh_0.2__ql_0.0 | |
| 119 | change_quantiles__f_agg_"var"__isabs_False__qh_0.4__ql_0.0 | |
| 120 | change_quantiles__f_agg_"var"__isabs_False__qh_0.4__ql_0.2 | |
| 121 | change_quantiles__f_agg_"var"__isabs_False__qh_0.6__ql_0.0 | |
| 122 | change_quantiles__f_agg_"var"__isabs_False__qh_0.6__ql_0.2 | |
| 123 | change_quantiles__f_agg_"var"__isabs_False__qh_0.6__ql_0.4 | |
| 124 | change_quantiles__f_agg_"var"__isabs_False__qh_0.8__ql_0.0 | |
| 125 | change_quantiles__f_agg_"var"__isabs_False__qh_0.8__ql_0.2 | |
| 126 | change_quantiles__f_agg_"var"__isabs_False__qh_0.8__ql_0.4 | |
| 127 | change_quantiles__f_agg_"var"__isabs_False__qh_0.8__ql_0.6 | |
| 128 | change_quantiles__f_agg_"var"__isabs_False__qh_1.0__ql_0.0 | |
| 129 | change_quantiles__f_agg_"var"__isabs_False__qh_1.0__ql_0.2 | |
| 130 | change_quantiles__f_agg_"var"__isabs_False__qh_1.0__ql_0.4 | |
| 131 | change_quantiles__f_agg_"var"__isabs_False__qh_1.0__ql_0.6 | |
| 132 | change_quantiles__f_agg_"var"__isabs_False__qh_1.0__ql_0.8 | |
| 133 | change_quantiles__f_agg_"var"__isabs_True__qh_0.2__ql_0.0 | |
| 134 | change_quantiles__f_agg_"var"__isabs_True__qh_0.4__ql_0.0 | |
| 135 | change_quantiles__f_agg_"var"__isabs_True__qh_0.4__ql_0.2 | |
| 136 | change_quantiles__f_agg_"var"__isabs_True__qh_0.6__ql_0.0 | |
| 137 | change_quantiles__f_agg_"var"__isabs_True__qh_0.6__ql_0.2 | |
| 138 | change_quantiles__f_agg_"var"__isabs_True__qh_0.6__ql_0.4 | |
| 139 | change_quantiles__f_agg_"var"__isabs_True__qh_0.8__ql_0.0 | |
| 140 | change_quantiles__f_agg_"var"__isabs_True__qh_0.8__ql_0.2 | |
| 141 | change_quantiles__f_agg_"var"__isabs_True__qh_0.8__ql_0.4 | |
| 142 | change_quantiles__f_agg_"var"__isabs_True__qh_0.8__ql_0.6 | |
| 143 | change_quantiles__f_agg_"var"__isabs_True__qh_1.0__ql_0.0 | |
| 144 | change_quantiles__f_agg_"var"__isabs_True__qh_1.0__ql_0.2 | |
| 145 | change_quantiles__f_agg_"var"__isabs_True__qh_1.0__ql_0.4 | |
| 146 | change_quantiles__f_agg_"var"__isabs_True__qh_1.0__ql_0.6 | |
| 147 | change_quantiles__f_agg_"var"__isabs_True__qh_1.0__ql_0.8 | |
| 148 | cid_ce__normalize_False | |
| 149 | cid_ce__normalize_True | |
| 150 | count_above__t_0 | |
| 151 | count_above_mean | |
| 152 | count_below__t_0 | |
| 153 | count_below_mean | |
| 154 | cwt_coefficients__coeff_0__w_10__widths_(2, 5, 10, 20) | |
| 155 | cwt_coefficients__coeff_0__w_20__widths_(2, 5, 10, 20) | |
| 156 | cwt_coefficients__coeff_0__w_2__widths_(2, 5, 10, 20) | |
| 157 | cwt_coefficients__coeff_0__w_5__widths_(2, 5, 10, 20) | |
| 158 | cwt_coefficients__coeff_10__w_10__widths_(2, 5, 10, 20) | |
| 159 | cwt_coefficients__coeff_10__w_20__widths_(2, 5, 10, 20) | |
| 160 | cwt_coefficients__coeff_10__w_2__widths_(2, 5, 10, 20) | |
| 161 | cwt_coefficients__coeff_10__w_5__widths_(2, 5, 10, 20) | |
| 162 | cwt_coefficients__coeff_11__w_10__widths_(2, 5, 10, 20) | |
| 163 | cwt_coefficients__coeff_11__w_20__widths_(2, 5, 10, 20) | |
| 164 | cwt_coefficients__coeff_11__w_2__widths_(2, 5, 10, 20) | |
| 165 | cwt_coefficients__coeff_11__w_5__widths_(2, 5, 10, 20) | |
| 166 | cwt_coefficients__coeff_12__w_10__widths_(2, 5, 10, 20) | |
| 167 | cwt_coefficients__coeff_12__w_20__widths_(2, 5, 10, 20) | |
| 168 | cwt_coefficients__coeff_12__w_2__widths_(2, 5, 10, 20) | |
| 169 | cwt_coefficients__coeff_12__w_5__widths_(2, 5, 10, 20) | |
| 170 | cwt_coefficients__coeff_13__w_10__widths_(2, 5, 10, 20) | |
| 171 | cwt_coefficients__coeff_13__w_20__widths_(2, 5, 10, 20) | |
| 172 | cwt_coefficients__coeff_13__w_2__widths_(2, 5, 10, 20) | |
| 173 | cwt_coefficients__coeff_13__w_5__widths_(2, 5, 10, 20) | |
| 174 | cwt_coefficients__coeff_14__w_10__widths_(2, 5, 10, 20) | |
| 175 | cwt_coefficients__coeff_14__w_20__widths_(2, 5, 10, 20) | |
| 176 | cwt_coefficients__coeff_14__w_2__widths_(2, 5, 10, 20) | |
| 177 | cwt_coefficients__coeff_14__w_5__widths_(2, 5, 10, 20) | |
| 178 | cwt_coefficients__coeff_1__w_10__widths_(2, 5, 10, 20) | |
| 179 | cwt_coefficients__coeff_1__w_20__widths_(2, 5, 10, 20) | |
| 180 | cwt_coefficients__coeff_1__w_2__widths_(2, 5, 10, 20) | |
| 181 | cwt_coefficients__coeff_1__w_5__widths_(2, 5, 10, 20) | |
| 182 | cwt_coefficients__coeff_2__w_10__widths_(2, 5, 10, 20) | |
| 183 | cwt_coefficients__coeff_2__w_20__widths_(2, 5, 10, 20) | |
| 184 | cwt_coefficients__coeff_2__w_2__widths_(2, 5, 10, 20) | |
| 185 | cwt_coefficients__coeff_2__w_5__widths_(2, 5, 10, 20) | |
| 186 | cwt_coefficients__coeff_3__w_10__widths_(2, 5, 10, 20) | |
| 187 | cwt_coefficients__coeff_3__w_20__widths_(2, 5, 10, 20) | |
| 188 | cwt_coefficients__coeff_3__w_2__widths_(2, 5, 10, 20) | |
| 189 | cwt_coefficients__coeff_3__w_5__widths_(2, 5, 10, 20) | |
| 190 | cwt_coefficients__coeff_4__w_10__widths_(2, 5, 10, 20) | |
| 191 | cwt_coefficients__coeff_4__w_20__widths_(2, 5, 10, 20) | |
| 192 | cwt_coefficients__coeff_4__w_2__widths_(2, 5, 10, 20) | |
| 193 | cwt_coefficients__coeff_4__w_5__widths_(2, 5, 10, 20) | |
| 194 | cwt_coefficients__coeff_5__w_10__widths_(2, 5, 10, 20) | |
| 195 | cwt_coefficients__coeff_5__w_20__widths_(2, 5, 10, 20) | |
| 196 | cwt_coefficients__coeff_5__w_2__widths_(2, 5, 10, 20) | |
| 197 | cwt_coefficients__coeff_5__w_5__widths_(2, 5, 10, 20) | |
| 198 | cwt_coefficients__coeff_6__w_10__widths_(2, 5, 10, 20) | |
| 199 | cwt_coefficients__coeff_6__w_20__widths_(2, 5, 10, 20) | |
| 200 | cwt_coefficients__coeff_6__w_2__widths_(2, 5, 10, 20) | |
| 201 | cwt_coefficients__coeff_6__w_5__widths_(2, 5, 10, 20) | |
| 202 | cwt_coefficients__coeff_7__w_10__widths_(2, 5, 10, 20) | |
| 203 | cwt_coefficients__coeff_7__w_20__widths_(2, 5, 10, 20) | |
| 204 | cwt_coefficients__coeff_7__w_2__widths_(2, 5, 10, 20) | |
| 205 | cwt_coefficients__coeff_7__w_5__widths_(2, 5, 10, 20) | |
| 206 | cwt_coefficients__coeff_8__w_10__widths_(2, 5, 10, 20) | |
| 207 | cwt_coefficients__coeff_8__w_20__widths_(2, 5, 10, 20) | |
| 208 | cwt_coefficients__coeff_8__w_2__widths_(2, 5, 10, 20) | |
| 209 | cwt_coefficients__coeff_8__w_5__widths_(2, 5, 10, 20) | |
| 210 | cwt_coefficients__coeff_9__w_10__widths_(2, 5, 10, 20) | |
| 211 | cwt_coefficients__coeff_9__w_20__widths_(2, 5, 10, 20) | |
| 212 | cwt_coefficients__coeff_9__w_2__widths_(2, 5, 10, 20) | |
| 213 | cwt_coefficients__coeff_9__w_5__widths_(2, 5, 10, 20) | |
| 214 | energy_ratio_by_chunks__num_segments_10__segment_focus_0 | |
| 215 | energy_ratio_by_chunks__num_segments_10__segment_focus_1 | |
| 216 | energy_ratio_by_chunks__num_segments_10__segment_focus_2 | |
| 217 | energy_ratio_by_chunks__num_segments_10__segment_focus_3 | |
| 218 | energy_ratio_by_chunks__num_segments_10__segment_focus_4 | |
| 219 | energy_ratio_by_chunks__num_segments_10__segment_focus_5 | |
| 220 | energy_ratio_by_chunks__num_segments_10__segment_focus_6 | |
| 221 | energy_ratio_by_chunks__num_segments_10__segment_focus_7 | |
| 222 | energy_ratio_by_chunks__num_segments_10__segment_focus_8 | |
| 223 | energy_ratio_by_chunks__num_segments_10__segment_focus_9 | |
| 224 | fft_aggregated__aggtype_"centroid" | |
| 225 | fft_aggregated__aggtype_"kurtosis" | |
| 226 | fft_aggregated__aggtype_"skew" | |
| 227 | fft_aggregated__aggtype_"variance" | |
| 228 | fft_coefficient__attr_"abs"__coeff_0 | |
| 229 | fft_coefficient__attr_"abs"__coeff_1 | |
| 230 | fft_coefficient__attr_"abs"__coeff_10 | |
| 231 | fft_coefficient__attr_"abs"__coeff_11 | |
| 232 | fft_coefficient__attr_"abs"__coeff_12 | |
| 233 | fft_coefficient__attr_"abs"__coeff_13 | |
| 234 | fft_coefficient__attr_"abs"__coeff_14 | |
| 235 | fft_coefficient__attr_"abs"__coeff_15 | |
| 236 | fft_coefficient__attr_"abs"__coeff_16 | |
| 237 | fft_coefficient__attr_"abs"__coeff_17 | |
| 238 | fft_coefficient__attr_"abs"__coeff_18 | |
| 239 | fft_coefficient__attr_"abs"__coeff_19 | |
| 240 | fft_coefficient__attr_"abs"__coeff_2 | |
| 241 | fft_coefficient__attr_"abs"__coeff_20 | |
| 242 | fft_coefficient__attr_"abs"__coeff_21 | |
| 243 | fft_coefficient__attr_"abs"__coeff_22 | |
| 244 | fft_coefficient__attr_"abs"__coeff_23 | |
| 245 | fft_coefficient__attr_"abs"__coeff_24 | |
| 246 | fft_coefficient__attr_"abs"__coeff_25 | |
| 247 | fft_coefficient__attr_"abs"__coeff_26 | |
| 248 | fft_coefficient__attr_"abs"__coeff_27 | |
| 249 | fft_coefficient__attr_"abs"__coeff_28 | |
| 250 | fft_coefficient__attr_"abs"__coeff_29 | |
| 251 | fft_coefficient__attr_"abs"__coeff_3 | |
| 252 | fft_coefficient__attr_"abs"__coeff_30 | |
| 253 | fft_coefficient__attr_"abs"__coeff_31 | |
| 254 | fft_coefficient__attr_"abs"__coeff_32 | |
| 255 | fft_coefficient__attr_"abs"__coeff_33 | |
| 256 | fft_coefficient__attr_"abs"__coeff_34 | |
| 257 | fft_coefficient__attr_"abs"__coeff_35 | |
| 258 | fft_coefficient__attr_"abs"__coeff_36 | |
| 259 | fft_coefficient__attr_"abs"__coeff_37 | |
| 260 | fft_coefficient__attr_"abs"__coeff_38 | |
| 261 | fft_coefficient__attr_"abs"__coeff_39 | |
| 262 | fft_coefficient__attr_"abs"__coeff_4 | |
| 263 | fft_coefficient__attr_"abs"__coeff_40 | |
| 264 | fft_coefficient__attr_"abs"__coeff_41 | |
| 265 | fft_coefficient__attr_"abs"__coeff_42 | |
| 266 | fft_coefficient__attr_"abs"__coeff_43 | |
| 267 | fft_coefficient__attr_"abs"__coeff_44 | |
| 268 | fft_coefficient__attr_"abs"__coeff_45 | |
| 269 | fft_coefficient__attr_"abs"__coeff_46 | |
| 270 | fft_coefficient__attr_"abs"__coeff_47 | |
| 271 | fft_coefficient__attr_"abs"__coeff_48 | |
| 272 | fft_coefficient__attr_"abs"__coeff_49 | |
| 273 | fft_coefficient__attr_"abs"__coeff_5 | |
| 274 | fft_coefficient__attr_"abs"__coeff_50 | |
| 275 | fft_coefficient__attr_"abs"__coeff_51 | |
| 276 | fft_coefficient__attr_"abs"__coeff_52 | |
| 277 | fft_coefficient__attr_"abs"__coeff_53 | |
| 278 | fft_coefficient__attr_"abs"__coeff_54 | |
| 279 | fft_coefficient__attr_"abs"__coeff_55 | |
| 280 | fft_coefficient__attr_"abs"__coeff_56 | |
| 281 | fft_coefficient__attr_"abs"__coeff_57 | |
| 282 | fft_coefficient__attr_"abs"__coeff_58 | |
| 283 | fft_coefficient__attr_"abs"__coeff_59 | |
| 284 | fft_coefficient__attr_"abs"__coeff_6 | |
| 285 | fft_coefficient__attr_"abs"__coeff_60 | |
| 286 | fft_coefficient__attr_"abs"__coeff_61 | |
| 287 | fft_coefficient__attr_"abs"__coeff_62 | |
| 288 | fft_coefficient__attr_"abs"__coeff_63 | |
| 289 | fft_coefficient__attr_"abs"__coeff_64 | |
| 290 | fft_coefficient__attr_"abs"__coeff_65 | |
| 291 | fft_coefficient__attr_"abs"__coeff_66 | |
| 292 | fft_coefficient__attr_"abs"__coeff_67 | |
| 293 | fft_coefficient__attr_"abs"__coeff_68 | |
| 294 | fft_coefficient__attr_"abs"__coeff_69 | |
| 295 | fft_coefficient__attr_"abs"__coeff_7 | |
| 296 | fft_coefficient__attr_"abs"__coeff_70 | |
| 297 | fft_coefficient__attr_"abs"__coeff_71 | |
| 298 | fft_coefficient__attr_"abs"__coeff_72 | |
| 299 | fft_coefficient__attr_"abs"__coeff_73 | |
| 300 | fft_coefficient__attr_"abs"__coeff_74 | |
| 301 | fft_coefficient__attr_"abs"__coeff_75 | |
| 302 | fft_coefficient__attr_"abs"__coeff_76 | |
| 303 | fft_coefficient__attr_"abs"__coeff_77 | |
| 304 | fft_coefficient__attr_"abs"__coeff_78 | |
| 305 | fft_coefficient__attr_"abs"__coeff_79 | |
| 306 | fft_coefficient__attr_"abs"__coeff_8 | |
| 307 | fft_coefficient__attr_"abs"__coeff_80 | |
| 308 | fft_coefficient__attr_"abs"__coeff_81 | |
| 309 | fft_coefficient__attr_"abs"__coeff_82 | |
| 310 | fft_coefficient__attr_"abs"__coeff_83 | |
| 311 | fft_coefficient__attr_"abs"__coeff_84 | |
| 312 | fft_coefficient__attr_"abs"__coeff_85 | |
| 313 | fft_coefficient__attr_"abs"__coeff_86 | |
| 314 | fft_coefficient__attr_"abs"__coeff_87 | |
| 315 | fft_coefficient__attr_"abs"__coeff_88 | |
| 316 | fft_coefficient__attr_"abs"__coeff_89 | |
| 317 | fft_coefficient__attr_"abs"__coeff_9 | |
| 318 | fft_coefficient__attr_"abs"__coeff_90 | |
| 319 | fft_coefficient__attr_"abs"__coeff_91 | |
| 320 | fft_coefficient__attr_"abs"__coeff_92 | |
| 321 | fft_coefficient__attr_"abs"__coeff_93 | |
| 322 | fft_coefficient__attr_"abs"__coeff_94 | |
| 323 | fft_coefficient__attr_"abs"__coeff_95 | |
| 324 | fft_coefficient__attr_"abs"__coeff_96 | |
| 325 | fft_coefficient__attr_"abs"__coeff_97 | |
| 326 | fft_coefficient__attr_"abs"__coeff_98 | |
| 327 | fft_coefficient__attr_"abs"__coeff_99 | |
| 328 | fft_coefficient__attr_"angle"__coeff_0 | |
| 329 | fft_coefficient__attr_"angle"__coeff_1 | |
| 330 | fft_coefficient__attr_"angle"__coeff_10 | |
| 331 | fft_coefficient__attr_"angle"__coeff_11 | |
| 332 | fft_coefficient__attr_"angle"__coeff_12 | |
| 333 | fft_coefficient__attr_"angle"__coeff_13 | |
| 334 | fft_coefficient__attr_"angle"__coeff_14 | |
| 335 | fft_coefficient__attr_"angle"__coeff_15 | |
| 336 | fft_coefficient__attr_"angle"__coeff_16 | |
| 337 | fft_coefficient__attr_"angle"__coeff_17 | |
| 338 | fft_coefficient__attr_"angle"__coeff_18 | |
| 339 | fft_coefficient__attr_"angle"__coeff_19 | |
| 340 | fft_coefficient__attr_"angle"__coeff_2 | |
| 341 | fft_coefficient__attr_"angle"__coeff_20 | |
| 342 | fft_coefficient__attr_"angle"__coeff_21 | |
| 343 | fft_coefficient__attr_"angle"__coeff_22 | |
| 344 | fft_coefficient__attr_"angle"__coeff_23 | |
| 345 | fft_coefficient__attr_"angle"__coeff_24 | |
| 346 | fft_coefficient__attr_"angle"__coeff_25 | |
| 347 | fft_coefficient__attr_"angle"__coeff_26 | |
| 348 | fft_coefficient__attr_"angle"__coeff_27 | |
| 349 | fft_coefficient__attr_"angle"__coeff_28 | |
| 350 | fft_coefficient__attr_"angle"__coeff_29 | |
| 351 | fft_coefficient__attr_"angle"__coeff_3 | |
| 352 | fft_coefficient__attr_"angle"__coeff_30 | |
| 353 | fft_coefficient__attr_"angle"__coeff_31 | |
| 354 | fft_coefficient__attr_"angle"__coeff_32 | |
| 355 | fft_coefficient__attr_"angle"__coeff_33 | |
| 356 | fft_coefficient__attr_"angle"__coeff_34 | |
| 357 | fft_coefficient__attr_"angle"__coeff_35 | |
| 358 | fft_coefficient__attr_"angle"__coeff_36 | |
| 359 | fft_coefficient__attr_"angle"__coeff_37 | |
| 360 | fft_coefficient__attr_"angle"__coeff_38 | |
| 361 | fft_coefficient__attr_"angle"__coeff_39 | |
| 362 | fft_coefficient__attr_"angle"__coeff_4 | |
| 363 | fft_coefficient__attr_"angle"__coeff_40 | |
| 364 | fft_coefficient__attr_"angle"__coeff_41 | |
| 365 | fft_coefficient__attr_"angle"__coeff_42 | |
| 366 | fft_coefficient__attr_"angle"__coeff_43 | |
| 367 | fft_coefficient__attr_"angle"__coeff_44 | |
| 368 | fft_coefficient__attr_"angle"__coeff_45 | |
| 369 | fft_coefficient__attr_"angle"__coeff_46 | |
| 370 | fft_coefficient__attr_"angle"__coeff_47 | |
| 371 | fft_coefficient__attr_"angle"__coeff_48 | |
| 372 | fft_coefficient__attr_"angle"__coeff_49 | |
| 373 | fft_coefficient__attr_"angle"__coeff_5 | |
| 374 | fft_coefficient__attr_"angle"__coeff_50 | |
| 375 | fft_coefficient__attr_"angle"__coeff_51 | |
| 376 | fft_coefficient__attr_"angle"__coeff_52 | |
| 377 | fft_coefficient__attr_"angle"__coeff_53 | |
| 378 | fft_coefficient__attr_"angle"__coeff_54 | |
| 379 | fft_coefficient__attr_"angle"__coeff_55 | |
| 380 | fft_coefficient__attr_"angle"__coeff_56 | |
| 381 | fft_coefficient__attr_"angle"__coeff_57 | |
| 382 | fft_coefficient__attr_"angle"__coeff_58 | |
| 383 | fft_coefficient__attr_"angle"__coeff_59 | |
| 384 | fft_coefficient__attr_"angle"__coeff_6 | |
| 385 | fft_coefficient__attr_"angle"__coeff_60 | |
| 386 | fft_coefficient__attr_"angle"__coeff_61 | |
| 387 | fft_coefficient__attr_"angle"__coeff_62 | |
| 388 | fft_coefficient__attr_"angle"__coeff_63 | |
| 389 | fft_coefficient__attr_"angle"__coeff_64 | |
| 390 | fft_coefficient__attr_"angle"__coeff_65 | |
| 391 | fft_coefficient__attr_"angle"__coeff_66 | |
| 392 | fft_coefficient__attr_"angle"__coeff_67 | |
| 393 | fft_coefficient__attr_"angle"__coeff_68 | |
| 394 | fft_coefficient__attr_"angle"__coeff_69 | |
| 395 | fft_coefficient__attr_"angle"__coeff_7 | |
| 396 | fft_coefficient__attr_"angle"__coeff_70 | |
| 397 | fft_coefficient__attr_"angle"__coeff_71 | |
| 398 | fft_coefficient__attr_"angle"__coeff_72 | |
| 399 | fft_coefficient__attr_"angle"__coeff_73 | |
| 400 | fft_coefficient__attr_"angle"__coeff_74 | |
| 401 | fft_coefficient__attr_"angle"__coeff_75 | |
| 402 | fft_coefficient__attr_"angle"__coeff_76 | |
| 403 | fft_coefficient__attr_"angle"__coeff_77 | |
| 404 | fft_coefficient__attr_"angle"__coeff_78 | |
| 405 | fft_coefficient__attr_"angle"__coeff_79 | |
| 406 | fft_coefficient__attr_"angle"__coeff_8 | |
| 407 | fft_coefficient__attr_"angle"__coeff_80 | |
| 408 | fft_coefficient__attr_"angle"__coeff_81 | |
| 409 | fft_coefficient__attr_"angle"__coeff_82 | |
| 410 | fft_coefficient__attr_"angle"__coeff_83 | |
| 411 | fft_coefficient__attr_"angle"__coeff_84 | |
| 412 | fft_coefficient__attr_"angle"__coeff_85 | |
| 413 | fft_coefficient__attr_"angle"__coeff_86 | |
| 414 | fft_coefficient__attr_"angle"__coeff_87 | |
| 415 | fft_coefficient__attr_"angle"__coeff_88 | |
| 416 | fft_coefficient__attr_"angle"__coeff_89 | |
| 417 | fft_coefficient__attr_"angle"__coeff_9 | |
| 418 | fft_coefficient__attr_"angle"__coeff_90 | |
| 419 | fft_coefficient__attr_"angle"__coeff_91 | |
| 420 | fft_coefficient__attr_"angle"__coeff_92 | |
| 421 | fft_coefficient__attr_"angle"__coeff_93 | |
| 422 | fft_coefficient__attr_"angle"__coeff_94 | |
| 423 | fft_coefficient__attr_"angle"__coeff_95 | |
| 424 | fft_coefficient__attr_"angle"__coeff_96 | |
| 425 | fft_coefficient__attr_"angle"__coeff_97 | |
| 426 | fft_coefficient__attr_"angle"__coeff_98 | |
| 427 | fft_coefficient__attr_"angle"__coeff_99 | |
| 428 | fft_coefficient__attr_"imag"__coeff_0 | |
| 429 | fft_coefficient__attr_"imag"__coeff_1 | |
| 430 | fft_coefficient__attr_"imag"__coeff_10 | |
| 431 | fft_coefficient__attr_"imag"__coeff_11 | |
| 432 | fft_coefficient__attr_"imag"__coeff_12 | |
| 433 | fft_coefficient__attr_"imag"__coeff_13 | |
| 434 | fft_coefficient__attr_"imag"__coeff_14 | |
| 435 | fft_coefficient__attr_"imag"__coeff_15 | |
| 436 | fft_coefficient__attr_"imag"__coeff_16 | |
| 437 | fft_coefficient__attr_"imag"__coeff_17 | |
| 438 | fft_coefficient__attr_"imag"__coeff_18 | |
| 439 | fft_coefficient__attr_"imag"__coeff_19 | |
| 440 | fft_coefficient__attr_"imag"__coeff_2 | |
| 441 | fft_coefficient__attr_"imag"__coeff_20 | |
| 442 | fft_coefficient__attr_"imag"__coeff_21 | |
| 443 | fft_coefficient__attr_"imag"__coeff_22 | |
| 444 | fft_coefficient__attr_"imag"__coeff_23 | |
| 445 | fft_coefficient__attr_"imag"__coeff_24 | |
| 446 | fft_coefficient__attr_"imag"__coeff_25 | |
| 447 | fft_coefficient__attr_"imag"__coeff_26 | |
| 448 | fft_coefficient__attr_"imag"__coeff_27 | |
| 449 | fft_coefficient__attr_"imag"__coeff_28 | |
| 450 | fft_coefficient__attr_"imag"__coeff_29 | |
| 451 | fft_coefficient__attr_"imag"__coeff_3 | |
| 452 | fft_coefficient__attr_"imag"__coeff_30 | |
| 453 | fft_coefficient__attr_"imag"__coeff_31 | |
| 454 | fft_coefficient__attr_"imag"__coeff_32 | |
| 455 | fft_coefficient__attr_"imag"__coeff_33 | |
| 456 | fft_coefficient__attr_"imag"__coeff_34 | |
| 457 | fft_coefficient__attr_"imag"__coeff_35 | |
| 458 | fft_coefficient__attr_"imag"__coeff_36 | |
| 459 | fft_coefficient__attr_"imag"__coeff_37 | |
| 460 | fft_coefficient__attr_"imag"__coeff_38 | |
| 461 | fft_coefficient__attr_"imag"__coeff_39 | |
| 462 | fft_coefficient__attr_"imag"__coeff_4 | |
| 463 | fft_coefficient__attr_"imag"__coeff_40 | |
| 464 | fft_coefficient__attr_"imag"__coeff_41 | |
| 465 | fft_coefficient__attr_"imag"__coeff_42 | |
| 466 | fft_coefficient__attr_"imag"__coeff_43 | |
| 467 | fft_coefficient__attr_"imag"__coeff_44 | |
| 468 | fft_coefficient__attr_"imag"__coeff_45 | |
| 469 | fft_coefficient__attr_"imag"__coeff_46 | |
| 470 | fft_coefficient__attr_"imag"__coeff_47 | |
| 471 | fft_coefficient__attr_"imag"__coeff_48 | |
| 472 | fft_coefficient__attr_"imag"__coeff_49 | |
| 473 | fft_coefficient__attr_"imag"__coeff_5 | |
| 474 | fft_coefficient__attr_"imag"__coeff_50 | |
| 475 | fft_coefficient__attr_"imag"__coeff_51 | |
| 476 | fft_coefficient__attr_"imag"__coeff_52 | |
| 477 | fft_coefficient__attr_"imag"__coeff_53 | |
| 478 | fft_coefficient__attr_"imag"__coeff_54 | |
| 479 | fft_coefficient__attr_"imag"__coeff_55 | |
| 480 | fft_coefficient__attr_"imag"__coeff_56 | |
| 481 | fft_coefficient__attr_"imag"__coeff_57 | |
| 482 | fft_coefficient__attr_"imag"__coeff_58 | |
| 483 | fft_coefficient__attr_"imag"__coeff_59 | |
| 484 | fft_coefficient__attr_"imag"__coeff_6 | |
| 485 | fft_coefficient__attr_"imag"__coeff_60 | |
| 486 | fft_coefficient__attr_"imag"__coeff_61 | |
| 487 | fft_coefficient__attr_"imag"__coeff_62 | |
| 488 | fft_coefficient__attr_"imag"__coeff_63 | |
| 489 | fft_coefficient__attr_"imag"__coeff_64 | |
| 490 | fft_coefficient__attr_"imag"__coeff_65 | |
| 491 | fft_coefficient__attr_"imag"__coeff_66 | |
| 492 | fft_coefficient__attr_"imag"__coeff_67 | |
| 493 | fft_coefficient__attr_"imag"__coeff_68 | |
| 494 | fft_coefficient__attr_"imag"__coeff_69 | |
| 495 | fft_coefficient__attr_"imag"__coeff_7 | |
| 496 | fft_coefficient__attr_"imag"__coeff_70 | |
| 497 | fft_coefficient__attr_"imag"__coeff_71 | |
| 498 | fft_coefficient__attr_"imag"__coeff_72 | |
| 499 | fft_coefficient__attr_"imag"__coeff_73 | |
| 500 | fft_coefficient__attr_"imag"__coeff_74 | |
| 501 | fft_coefficient__attr_"imag"__coeff_75 | |
| 502 | fft_coefficient__attr_"imag"__coeff_76 | |
| 503 | fft_coefficient__attr_"imag"__coeff_77 | |
| 504 | fft_coefficient__attr_"imag"__coeff_78 | |
| 505 | fft_coefficient__attr_"imag"__coeff_79 | |
| 506 | fft_coefficient__attr_"imag"__coeff_8 | |
| 507 | fft_coefficient__attr_"imag"__coeff_80 | |
| 508 | fft_coefficient__attr_"imag"__coeff_81 | |
| 509 | fft_coefficient__attr_"imag"__coeff_82 | |
| 510 | fft_coefficient__attr_"imag"__coeff_83 | |
| 511 | fft_coefficient__attr_"imag"__coeff_84 | |
| 512 | fft_coefficient__attr_"imag"__coeff_85 | |
| 513 | fft_coefficient__attr_"imag"__coeff_86 | |
| 514 | fft_coefficient__attr_"imag"__coeff_87 | |
| 515 | fft_coefficient__attr_"imag"__coeff_88 | |
| 516 | fft_coefficient__attr_"imag"__coeff_89 | |
| 517 | fft_coefficient__attr_"imag"__coeff_9 | |
| 518 | fft_coefficient__attr_"imag"__coeff_90 | |
| 519 | fft_coefficient__attr_"imag"__coeff_91 | |
| 520 | fft_coefficient__attr_"imag"__coeff_92 | |
| 521 | fft_coefficient__attr_"imag"__coeff_93 | |
| 522 | fft_coefficient__attr_"imag"__coeff_94 | |
| 523 | fft_coefficient__attr_"imag"__coeff_95 | |
| 524 | fft_coefficient__attr_"imag"__coeff_96 | |
| 525 | fft_coefficient__attr_"imag"__coeff_97 | |
| 526 | fft_coefficient__attr_"imag"__coeff_98 | |
| 527 | fft_coefficient__attr_"imag"__coeff_99 | |
| 528 | fft_coefficient__attr_"real"__coeff_0 | |
| 529 | fft_coefficient__attr_"real"__coeff_1 | |
| 530 | fft_coefficient__attr_"real"__coeff_10 | |
| 531 | fft_coefficient__attr_"real"__coeff_11 | |
| 532 | fft_coefficient__attr_"real"__coeff_12 | |
| 533 | fft_coefficient__attr_"real"__coeff_13 | |
| 534 | fft_coefficient__attr_"real"__coeff_14 | |
| 535 | fft_coefficient__attr_"real"__coeff_15 | |
| 536 | fft_coefficient__attr_"real"__coeff_16 | |
| 537 | fft_coefficient__attr_"real"__coeff_17 | |
| 538 | fft_coefficient__attr_"real"__coeff_18 | |
| 539 | fft_coefficient__attr_"real"__coeff_19 | |
| 540 | fft_coefficient__attr_"real"__coeff_2 | |
| 541 | fft_coefficient__attr_"real"__coeff_20 | |
| 542 | fft_coefficient__attr_"real"__coeff_21 | |
| 543 | fft_coefficient__attr_"real"__coeff_22 | |
| 544 | fft_coefficient__attr_"real"__coeff_23 | |
| 545 | fft_coefficient__attr_"real"__coeff_24 | |
| 546 | fft_coefficient__attr_"real"__coeff_25 | |
| 547 | fft_coefficient__attr_"real"__coeff_26 | |
| 548 | fft_coefficient__attr_"real"__coeff_27 | |
| 549 | fft_coefficient__attr_"real"__coeff_28 | |
| 550 | fft_coefficient__attr_"real"__coeff_29 | |
| 551 | fft_coefficient__attr_"real"__coeff_3 | |
| 552 | fft_coefficient__attr_"real"__coeff_30 | |
| 553 | fft_coefficient__attr_"real"__coeff_31 | |
| 554 | fft_coefficient__attr_"real"__coeff_32 | |
| 555 | fft_coefficient__attr_"real"__coeff_33 | |
| 556 | fft_coefficient__attr_"real"__coeff_34 | |
| 557 | fft_coefficient__attr_"real"__coeff_35 | |
| 558 | fft_coefficient__attr_"real"__coeff_36 | |
| 559 | fft_coefficient__attr_"real"__coeff_37 | |
| 560 | fft_coefficient__attr_"real"__coeff_38 | |
| 561 | fft_coefficient__attr_"real"__coeff_39 | |
| 562 | fft_coefficient__attr_"real"__coeff_4 | |
| 563 | fft_coefficient__attr_"real"__coeff_40 | |
| 564 | fft_coefficient__attr_"real"__coeff_41 | |
| 565 | fft_coefficient__attr_"real"__coeff_42 | |
| 566 | fft_coefficient__attr_"real"__coeff_43 | |
| 567 | fft_coefficient__attr_"real"__coeff_44 | |
| 568 | fft_coefficient__attr_"real"__coeff_45 | |
| 569 | fft_coefficient__attr_"real"__coeff_46 | |
| 570 | fft_coefficient__attr_"real"__coeff_47 | |
| 571 | fft_coefficient__attr_"real"__coeff_48 | |
| 572 | fft_coefficient__attr_"real"__coeff_49 | |
| 573 | fft_coefficient__attr_"real"__coeff_5 | |
| 574 | fft_coefficient__attr_"real"__coeff_50 | |
| 575 | fft_coefficient__attr_"real"__coeff_51 | |
| 576 | fft_coefficient__attr_"real"__coeff_52 | |
| 577 | fft_coefficient__attr_"real"__coeff_53 | |
| 578 | fft_coefficient__attr_"real"__coeff_54 | |
| 579 | fft_coefficient__attr_"real"__coeff_55 | |
| 580 | fft_coefficient__attr_"real"__coeff_56 | |
| 581 | fft_coefficient__attr_"real"__coeff_57 | |
| 582 | fft_coefficient__attr_"real"__coeff_58 | |
| 583 | fft_coefficient__attr_"real"__coeff_59 | |
| 584 | fft_coefficient__attr_"real"__coeff_6 | |
| 585 | fft_coefficient__attr_"real"__coeff_60 | |
| 586 | fft_coefficient__attr_"real"__coeff_61 | |
| 587 | fft_coefficient__attr_"real"__coeff_62 | |
| 588 | fft_coefficient__attr_"real"__coeff_63 | |
| 589 | fft_coefficient__attr_"real"__coeff_64 | |
| 590 | fft_coefficient__attr_"real"__coeff_65 | |
| 591 | fft_coefficient__attr_"real"__coeff_66 | |
| 592 | fft_coefficient__attr_"real"__coeff_67 | |
| 593 | fft_coefficient__attr_"real"__coeff_68 | |
| 594 | fft_coefficient__attr_"real"__coeff_69 | |
| 595 | fft_coefficient__attr_"real"__coeff_7 | |
| 596 | fft_coefficient__attr_"real"__coeff_70 | |
| 597 | fft_coefficient__attr_"real"__coeff_71 | |
| 598 | fft_coefficient__attr_"real"__coeff_72 | |
| 599 | fft_coefficient__attr_"real"__coeff_73 | |
| 600 | fft_coefficient__attr_"real"__coeff_74 | |
| 601 | fft_coefficient__attr_"real"__coeff_75 | |
| 602 | fft_coefficient__attr_"real"__coeff_76 | |
| 603 | fft_coefficient__attr_"real"__coeff_77 | |
| 604 | fft_coefficient__attr_"real"__coeff_78 | |
| 605 | fft_coefficient__attr_"real"__coeff_79 | |
| 606 | fft_coefficient__attr_"real"__coeff_8 | |
| 607 | fft_coefficient__attr_"real"__coeff_80 | |
| 608 | fft_coefficient__attr_"real"__coeff_81 | |
| 609 | fft_coefficient__attr_"real"__coeff_82 | |
| 610 | fft_coefficient__attr_"real"__coeff_83 | |
| 611 | fft_coefficient__attr_"real"__coeff_84 | |
| 612 | fft_coefficient__attr_"real"__coeff_85 | |
| 613 | fft_coefficient__attr_"real"__coeff_86 | |
| 614 | fft_coefficient__attr_"real"__coeff_87 | |
| 615 | fft_coefficient__attr_"real"__coeff_88 | |
| 616 | fft_coefficient__attr_"real"__coeff_89 | |
| 617 | fft_coefficient__attr_"real"__coeff_9 | |
| 618 | fft_coefficient__attr_"real"__coeff_90 | |
| 619 | fft_coefficient__attr_"real"__coeff_91 | |
| 620 | fft_coefficient__attr_"real"__coeff_92 | |
| 621 | fft_coefficient__attr_"real"__coeff_93 | |
| 622 | fft_coefficient__attr_"real"__coeff_94 | |
| 623 | fft_coefficient__attr_"real"__coeff_95 | |
| 624 | fft_coefficient__attr_"real"__coeff_96 | |
| 625 | fft_coefficient__attr_"real"__coeff_97 | |
| 626 | fft_coefficient__attr_"real"__coeff_98 | |
| 627 | fft_coefficient__attr_"real"__coeff_99 | |
| 628 | first_location_of_maximum | |
| 629 | first_location_of_minimum | |
| 630 | fourier_entropy__bins_10 | |
| 631 | fourier_entropy__bins_100 | |
| 632 | fourier_entropy__bins_2 | |
| 633 | fourier_entropy__bins_3 | |
| 634 | fourier_entropy__bins_5 | |
| 635 | friedrich_coefficients__coeff_0__m_3__r_30 | |
| 636 | friedrich_coefficients__coeff_1__m_3__r_30 | |
| 637 | friedrich_coefficients__coeff_2__m_3__r_30 | |
| 638 | friedrich_coefficients__coeff_3__m_3__r_30 | |
| 639 | has_duplicate | |
| 640 | has_duplicate_max | |
| 641 | has_duplicate_min | |
| 642 | index_mass_quantile__q_0.1 | |
| 643 | index_mass_quantile__q_0.2 | |
| 644 | index_mass_quantile__q_0.3 | |
| 645 | index_mass_quantile__q_0.4 | |
| 646 | index_mass_quantile__q_0.6 | |
| 647 | index_mass_quantile__q_0.7 | |
| 648 | index_mass_quantile__q_0.8 | |
| 649 | index_mass_quantile__q_0.9 | |
| 650 | kurtosis |  |
| 651 | large_standard_deviation__r_0.05 | |
| 652 | large_standard_deviation__r_0.1 | |
| 653 | large_standard_deviation__r_0.15000000000000002 | |
| 654 | large_standard_deviation__r_0.2 | |
| 655 | large_standard_deviation__r_0.25 | |
| 656 | large_standard_deviation__r_0.30000000000000004 | |
| 657 | large_standard_deviation__r_0.35000000000000003 | |
| 658 | large_standard_deviation__r_0.4 | |
| 659 | large_standard_deviation__r_0.45 | |
| 660 | large_standard_deviation__r_0.5 | |
| 661 | large_standard_deviation__r_0.55 | |
| 662 | large_standard_deviation__r_0.6000000000000001 | |
| 663 | large_standard_deviation__r_0.65 | |
| 664 | large_standard_deviation__r_0.7000000000000001 | |
| 665 | large_standard_deviation__r_0.75 | |
| 666 | large_standard_deviation__r_0.8 | |
| 667 | large_standard_deviation__r_0.8500000000000001 | |
| 668 | large_standard_deviation__r_0.9 | |
| 669 | large_standard_deviation__r_0.9500000000000001 | |
| 670 | last_location_of_maximum | |
| 671 | last_location_of_minimum | |
| 672 | lempel_ziv_complexity__bins_10 | |
| 673 | lempel_ziv_complexity__bins_100 | |
| 674 | lempel_ziv_complexity__bins_2 | |
| 675 | lempel_ziv_complexity__bins_3 | |
| 676 | lempel_ziv_complexity__bins_5 | |
| 677 | length |  |
| 678 | linear_trend__attr_"intercept" | |
| 679 | linear_trend__attr_"pvalue" | |
| 680 | linear_trend__attr_"rvalue" | |
| 681 | linear_trend__attr_"slope" | |
| 682 | linear_trend__attr_"stderr" | |
| 683 | longest_strike_above_mean | |
| 684 | longest_strike_below_mean | |
| 685 | max_langevin_fixed_point__m_3__r_30 | |
| 686 | maximum |  |
| 687 | mean |  |
| 688 | mean_abs_change | |
| 689 | mean_change | |
| 690 | mean_n_absolute_max__number_of_maxima_7 | |
| 691 | mean_second_derivative_central | |
| 692 | median |  |
| 693 | minimum |  |
| 694 | number_crossing_m__m_-1 | |
| 695 | number_crossing_m__m_0 | |
| 696 | number_crossing_m__m_1 | |
| 697 | number_cwt_peaks__n_1 | |
| 698 | number_cwt_peaks__n_5 | |
| 699 | number_peaks__n_1 | |
| 700 | number_peaks__n_10 | |
| 701 | number_peaks__n_3 | |
| 702 | number_peaks__n_5 | |
| 703 | number_peaks__n_50 | |
| 704 | partial_autocorrelation__lag_0 | |
| 705 | partial_autocorrelation__lag_1 | |
| 706 | partial_autocorrelation__lag_2 | |
| 707 | partial_autocorrelation__lag_3 | |
| 708 | partial_autocorrelation__lag_4 | |
| 709 | partial_autocorrelation__lag_5 | |
| 710 | partial_autocorrelation__lag_6 | |
| 711 | partial_autocorrelation__lag_7 | |
| 712 | partial_autocorrelation__lag_8 | |
| 713 | partial_autocorrelation__lag_9 | |
| 714 | percentage_of_reoccurring_datapoints_to_all_datapoints | |
| 715 | percentage_of_reoccurring_values_to_all_values | |
| 716 | permutation_entropy__dimension_3__tau_1 | |
| 717 | permutation_entropy__dimension_4__tau_1 | |
| 718 | permutation_entropy__dimension_5__tau_1 | |
| 719 | permutation_entropy__dimension_6__tau_1 | |
| 720 | permutation_entropy__dimension_7__tau_1 | |
| 721 | quantile__q_0.1 | |
| 722 | quantile__q_0.2 | |
| 723 | quantile__q_0.3 | |
| 724 | quantile__q_0.4 | |
| 725 | quantile__q_0.6 | |
| 726 | quantile__q_0.7 | |
| 727 | quantile__q_0.8 | |
| 728 | quantile__q_0.9 | |
| 729 | query_similarity_count__query_None__threshold_0.0 | |
| 730 | range_count__max_0__min_-1000000000000.0 | |
| 731 | range_count__max_1000000000000.0__min_0 | |
| 732 | range_count__max_1__min_-1 | |
| 733 | ratio_beyond_r_sigma__r_0.5 | |
| 734 | ratio_beyond_r_sigma__r_1 | |
| 735 | ratio_beyond_r_sigma__r_1.5 | |
| 736 | ratio_beyond_r_sigma__r_10 | |
| 737 | ratio_beyond_r_sigma__r_2 | |
| 738 | ratio_beyond_r_sigma__r_2.5 | |
| 739 | ratio_beyond_r_sigma__r_3 | |
| 740 | ratio_beyond_r_sigma__r_5 | |
| 741 | ratio_beyond_r_sigma__r_6 | |
| 742 | ratio_beyond_r_sigma__r_7 | |
| 743 | ratio_value_number_to_time_series_length | |
| 744 | root_mean_square | |
| 745 | sample_entropy | |
| 746 | skewness |  |
| 747 | spkt_welch_density__coeff_2 | |
| 748 | spkt_welch_density__coeff_5 | |
| 749 | spkt_welch_density__coeff_8 | |
| 750 | standard_deviation | |
| 751 | sum_of_reoccurring_data_points | |
| 752 | sum_of_reoccurring_values | |
| 753 | sum_values |  |
| 754 | symmetry_looking__r_0.0 | |
| 755 | symmetry_looking__r_0.05 | |
| 756 | symmetry_looking__r_0.1 | |
| 757 | symmetry_looking__r_0.15000000000000002 | |
| 758 | symmetry_looking__r_0.2 | |
| 759 | symmetry_looking__r_0.25 | |
| 760 | symmetry_looking__r_0.30000000000000004 | |
| 761 | symmetry_looking__r_0.35000000000000003 | |
| 762 | symmetry_looking__r_0.4 | |
| 763 | symmetry_looking__r_0.45 | |
| 764 | symmetry_looking__r_0.5 | |
| 765 | symmetry_looking__r_0.55 | |
| 766 | symmetry_looking__r_0.6000000000000001 | |
| 767 | symmetry_looking__r_0.65 | |
| 768 | symmetry_looking__r_0.7000000000000001 | |
| 769 | symmetry_looking__r_0.75 | |
| 770 | symmetry_looking__r_0.8 | |
| 771 | symmetry_looking__r_0.8500000000000001 | |
| 772 | symmetry_looking__r_0.9 | |
| 773 | symmetry_looking__r_0.9500000000000001 | |
| 774 | time_reversal_asymmetry_statistic__lag_1 | |
| 775 | time_reversal_asymmetry_statistic__lag_2 | |
| 776 | time_reversal_asymmetry_statistic__lag_3 | |
| 777 | value_count__value_-1 | |
| 778 | value_count__value_0 | |
| 779 | value_count__value_1 | |
| 780 | variance |  |
| 781 | variance_larger_than_standard_deviation | |
| 782 | variation_coefficient | |

## **Supplementary Table 2: Extracted digital timeseries features**

The 783 feature extracted with tsfresh for each of the 14 digital measures are shown.

|  |  | **n** | **r** | **CI95%** | **p-val** | **BF10** | **power** | **FDR corrected p-val** |
| --- | --- | --- | --- | --- | --- | --- | --- | --- |
| DaTscan minimum\nputamen SBR | CSF alpha-synuclein\nSAA Fmax mean | 109 | -0.36 | [-0.51, -0.18] | 1.21E-04 | 175.917 | 0.97 | 2.41E-04 |
|  | MDS | 109 | -0.26 | [-0.43, -0.08] | 5.45E-03 | 5.409 | 0.80 | 6.81E-03 |
|  | MDS restricted | 109 | -0.20 | [-0.37, -0.01] | 3.92E-02 | 0.977 | 0.54 | 3.92E-02 |
|  | digital | 109 | -0.32 | [-0.48, -0.14] | 6.64E-04 | 36.35 | 0.93 | 9.49E-04 |
| CSF alpha-synuclein\nSAA Fmax mean | MDS | 109 | 0.52 | [0.36, 0.64] | 0.00E+00 | 1533000 | 1.00 | 0.00E+00 |
|  | MDS restricted | 109 | 0.43 | [0.26, 0.57] | 4.00E-06 | 4401.425 | 1.00 | 1.30E-05 |
|  | digital | 109 | 0.20 | [0.01, 0.38] | 3.57E-02 | 1.055 | 0.56 | 3.92E-02 |
| MDS | MDS restricted | 109 | 0.96 | [0.95, 0.97] | 0.00E+00 | 2.36E+58 | 1.00 | 0.00E+00 |
|  | digital | 109 | 0.36 | [0.18, 0.51] | 1.46E-04 | 147.297 | 0.97 | 2.43E-04 |
| MDS restricted | digital | 109 | 0.37 | [0.2, 0.52] | 6.60E-05 | 307.826 | 0.98 | 1.65E-04 |

## **Supplementary Table 3: Correlation of risk scores and biological measures**

We investigated the correlation of the risk scores (digital and MDS) and the biological measures with Pearson correlation. The statistics for each pair are shown with the sample size (n), r coefficient, 95% Confidence Interval (CI), uncorrected p-value, Bayes Factor (BF) 10, and power. After 0.05-Bonferroni adjustment, a p-value < 0.005 is significant.

| **marker** | **T** | **dof** | **alternative** | **p-val** | **CI95%** | **cohen-d** | **BF10** | **power** | **N_cases** | **N_controls** | **FDR-corrected** |
| --- | --- | --- | --- | --- | --- | --- | --- | --- | --- | --- | --- |
| male | 2.24 | 87.88 | two-sided | 2.77E-02 | [0.02 0.26] | 0.43 | 1.898 | 0.58 | 40 | 69 | 9.70E-02 |
| rbd_test | 1.37 | 18.78 | two-sided | 1.87E-01 | [-0.1 0.48] | 0.55 | 0.714 | 0.28 | 12 | 15 | 2.45E-01 |
| HYPOSMIA | 2.81 | 32.55 | two-sided | 8.32E-03 | [0.06 0.36] | 0.67 | 6.886 | 0.79 | 22 | 87 | 3.88E-02 |
| constipation_ | 0.06 | 45.32 | two-sided | 9.54E-01 | [-0.13 0.14] | 0.01 | 0.236 | 0.05 | 25 | 84 | 9.54E-01 |
| DepressionAnxiety_ | 3.27 | 92.71 | two-sided | 1.53E-03 | [0.08 0.31] | 0.62 | 21.175 | 0.87 | 40 | 69 | 1.07E-02 |
| ExcessiveDaytimeSleepiness_ | 0.54 | 46.56 | two-sided | 5.90E-01 | [-0.1 0.17] | 0.11 | 0.265 | 0.08 | 26 | 83 | 6.89E-01 |
| UPDRS>6 | 5.63 | 65.09 | two-sided | 4.16E-07 | [0.21 0.44] | 1.11 | 63230 | 1.00 | 27 | 70 | 5.83E-06 |
| ErectileDysfunction_ | 1.77 | 36.82 | two-sided | 8.47E-02 | [-0.02 0.33] | 0.53 | 1.064 | 0.35 | 15 | 25 | 1.56E-01 |
| UrinaryDysfunction_ | 1.34 | 28.38 | two-sided | 1.92E-01 | [-0.05 0.25] | 0.31 | 0.546 | 0.23 | 19 | 90 | 2.45E-01 |
| OrthostaticHypotension_ | 1.77 | 23.08 | two-sided | 8.91E-02 | [-0.02 0.31] | 0.45 | 0.989 | 0.40 | 17 | 92 | 1.56E-01 |
| 1st_degree_family_history_ | -0.33 | 77.06 | two-sided | 7.40E-01 | [-0.15 0.11] | 0.07 | 0.235 | 0.06 | 56 | 36 | 7.97E-01 |
| cognitive_impairment | 1.90 | 34.93 | two-sided | 6.54E-02 | [-0.01 0.28] | 0.43 | 1.145 | 0.43 | 22 | 87 | 1.53E-01 |
| GBA | -2.08 | 99.69 | two-sided | 3.99E-02 | [-0.24 -0.01] | 0.39 | 1.405 | 0.51 | 66 | 43 | 1.12E-01 |
| LRRK2 | 1.38 | 90.58 | two-sided | 1.72E-01 | [-0.04 0.2 ] | 0.26 | 0.49 | 0.25 | 38 | 71 | 2.45E-01 |

## **Supplementary Table 4: Differences in digital score between risk factor and prodromal symptom carriers**

The results of the two-sided T-tests are shown as the t-statistic, p-value, and number of individuals for each pair of risk factor/prodromal symptom and risk score.

|  |  | **mean** | **std** | **N** |
| --- | --- | --- | --- | --- |
| risk score | group |  |  |  |
| MDS | LRRK2 | 0.11 | 0.24 | 38 |
|  | GBA | 0.12 | 0.26 | 66 |
|  | RBD |  |  | 0 |
|  | hyposmia | 0.52 | 0.43 | 22 |
|  | DaT+ | 0.60 | 0.41 | 7 |
|  | SAA+ | 0.57 | 0.44 | 14 |
| MDS restricted | LRRK2 | 0.11 | 0.25 | 38 |
|  | GBA | 0.13 | 0.28 | 66 |
|  | RBD |  |  | 0 |
|  | hyposmia | 0.50 | 0.41 | 22 |
|  | DaT+ | 0.35 | 0.42 | 7 |
|  | SAA+ | 0.50 | 0.43 | 14 |
| digital | LRRK2 | 0.60 | 0.28 | 38 |
|  | GBA | 0.49 | 0.34 | 66 |
|  | RBD |  |  | 0 |
|  | hyposmia | 0.71 | 0.31 | 22 |
|  | DaT+ | 0.60 | 0.32 | 7 |
|  | SAA+ | 0.74 | 0.31 | 14 |

## **Supplementary Table 5: Mean risk score for each at-risk group**

For each at-risk group (LRRK2, GBA, hyposmia, RBD, DaT+, SAA+) we show the mean and standard deviation of the MDS, restricted MDS, and digital risk score.

| **Marker** | **True outcome** | **Statistic** | | | | | | |
| --- | --- | --- | --- | --- | --- | --- | --- | --- |
| statistic | SAA+ | TN | FP | FN | TP | precision | recall | fscore |
| MDS |  | 92 | 3 | 8 | 6 | 0.67 | 0.43 | 0.52 |
| MDS restricted |  | 91 | 4 | 8 | 6 | 0.60 | 0.43 | 0.50 |
| Hyposmia |  | 83 | 12 | 4 | 10 | 0.45 | 0.71 | 0.56 |
| digital |  | 49 | 46 | 4 | 10 | 0.18 | 0.71 | 0.29 |
| digital+hyposmia |  | 45 | 50 | 2 | 12 | 0.19 | 0.86 | 0.32 |
| SAA |  | 95 | 0 | 0 | 14 | 1.00 | 1.00 | 1.00 |
| DaTscan |  | 92 | 3 | 10 | 4 | 0.57 | 0.29 | 0.38 |
| MDS | Dat+ | 96 | 6 | 4 | 3 | 0.33 | 0.43 | 0.38 |
| MDS restricted |  | 93 | 9 | 6 | 1 | 0.10 | 0.14 | 0.12 |
| Hyposmia |  | 84 | 18 | 3 | 4 | 0.18 | 0.57 | 0.28 |
| digital |  | 49 | 53 | 4 | 3 | 0.05 | 0.43 | 0.10 |
| digital+hyposmia |  | 44 | 58 | 3 | 4 | 0.06 | 0.57 | 0.12 |
| SAA |  | 92 | 10 | 3 | 4 | 0.29 | 0.57 | 0.38 |
| DaTscan |  | ### | 0 | 0 | 7 | 1.00 | 1.00 | 1.00 |
| MDS | SAA+ or Dat+ | 89 | 3 | 11 | 6 | 0.67 | 0.35 | 0.46 |
| MDS restricted |  | 88 | 4 | 11 | 6 | 0.60 | 0.35 | 0.44 |
| Hyposmia |  | 80 | 12 | 7 | 10 | 0.45 | 0.59 | 0.51 |
| digital |  | 46 | 46 | 7 | 10 | 0.18 | 0.59 | 0.27 |
| digital+hyposmia |  | 42 | 50 | 5 | 12 | 0.19 | 0.71 | 0.30 |
| SAA |  | 92 | 0 | 3 | 14 | 1.00 | 0.82 | 0.90 |
| DaTscan |  | 92 | 0 | 10 | 7 | 1.00 | 0.41 | 0.58 |

## **Supplementary Table 6: Performance of the risk scores/markers in identifying DaTscan and alpha-synuclein positivity**

The table displays the performance analysis of different risk scores or presence of prodromal markers for identifying SAA positivity, DaTscan positivity, or their combination. This is measured in true negatives (TN), false negatives (FN), true positives (TP), false positives (FP), as well as precision, recall, and F1 score.

|  | prediction | digital | digital | digital | hyposmia | hyposmia | hyposmia | MDS restricted | MDS resctricted | MDS restricted |
| --- | --- | --- | --- | --- | --- | --- | --- | --- | --- | --- |
|  | TRUE outcome | SAA | DaT+ | SAA_DaT+ | SAA | DaT+ | DaT+ | SAA | DaT+ | SAA_DaT+ |
| Perfomance | T | -4.02 | -1.72 | -3.76 | -4.39 | -1.52 | -3.98 | -5.21 | -8.52 | -5.87 |
|  | dof | 9.67 | 2.08 | 10.01 | 9.12 | 3.36 | 10.02 | 11.03 | 5.00 | 9.24 |
|  | alternative | two-sided | two-sided | two-sided | two-sided | two-sided | two-sided | two-sided | two-sided | two-sided |
|  | p-val | 2.59E-03 | 2.23E-01 | 3.74E-03 | 1.69E-03 | 2.16E-01 | 2.58E-03 | 2.86E-04 | 3.67E-04 | 2.15E-04 |
|  | CI95% | [-22.88 -6.52] | [-50.03 20.69] | [-22.05 -5.63] | [-23.32 -7.48] | [-32.66 10.66] | [-22.34 -6.32] | [-26.96 -10.96] | [-1.98 13.31] | [-26.38 -11.74] |
|  | cohen-d | 1.49 | 1.55 | 1.56 | 1.61 | 1.00 | 1.65 | 2.81 | 3.48 | 3.10 |
|  | BF10 | 18.114 | 1.091 | 17.723 | 29.652 | 0.964 | 25.362 | 96.385 | 87.067 | 495.997 |
|  | power | 0.64 | 0.38 | 0.84 | 0.70 | 0.19 | 0.88 | 1.00 | 1.00 | 1.00 |
|  | mean FN | 1.00 | 3.00 | 1.86 | 0.50 | 3.00 | 1.57 | 3.38 | 5.67 | 3.27 |
|  | mean TP | 15.70 | 17.67 | 15.70 | 15.90 | 14.00 | 15.90 | 22.33 | 31.00 | 22.33 |
|  | std FN | 1.41 | 2.45 | 2.27 | 0.58 | 3.00 | 2.23 | 6.80 | 7.28 | 5.85 |
|  | std TP | 11.33 | 14.64 | 11.33 | 11.06 | 14.02 | 11.06 | 6.68 |  | 6.68 |
|  | N FN | 4 | 4 | 7 | 4 | 3 | 7 | 8 | 6 | 11 |
|  | N TP | 10 | 3 | 10 | 10 | 4 | 10 | 6 | 1 | 6 |

## **Supplementary Table 7: Differences in UPDRS III between individuals at biological and pathological risk identified and missed by digital risk score**

We report the statistical results of the comparison between false negatives and true positives for UPDRS III scores with two-sided Welch t-tests. We report the t-statistic, degrees of freedom, p-value, 95% Confidence Interval, and cohen’s d. This is reported for the comparison between the digital risk, restricted prodromal risk, and hyposmia with SAA or DaTscan positivity as the true outcome.

# Supplemental Figures


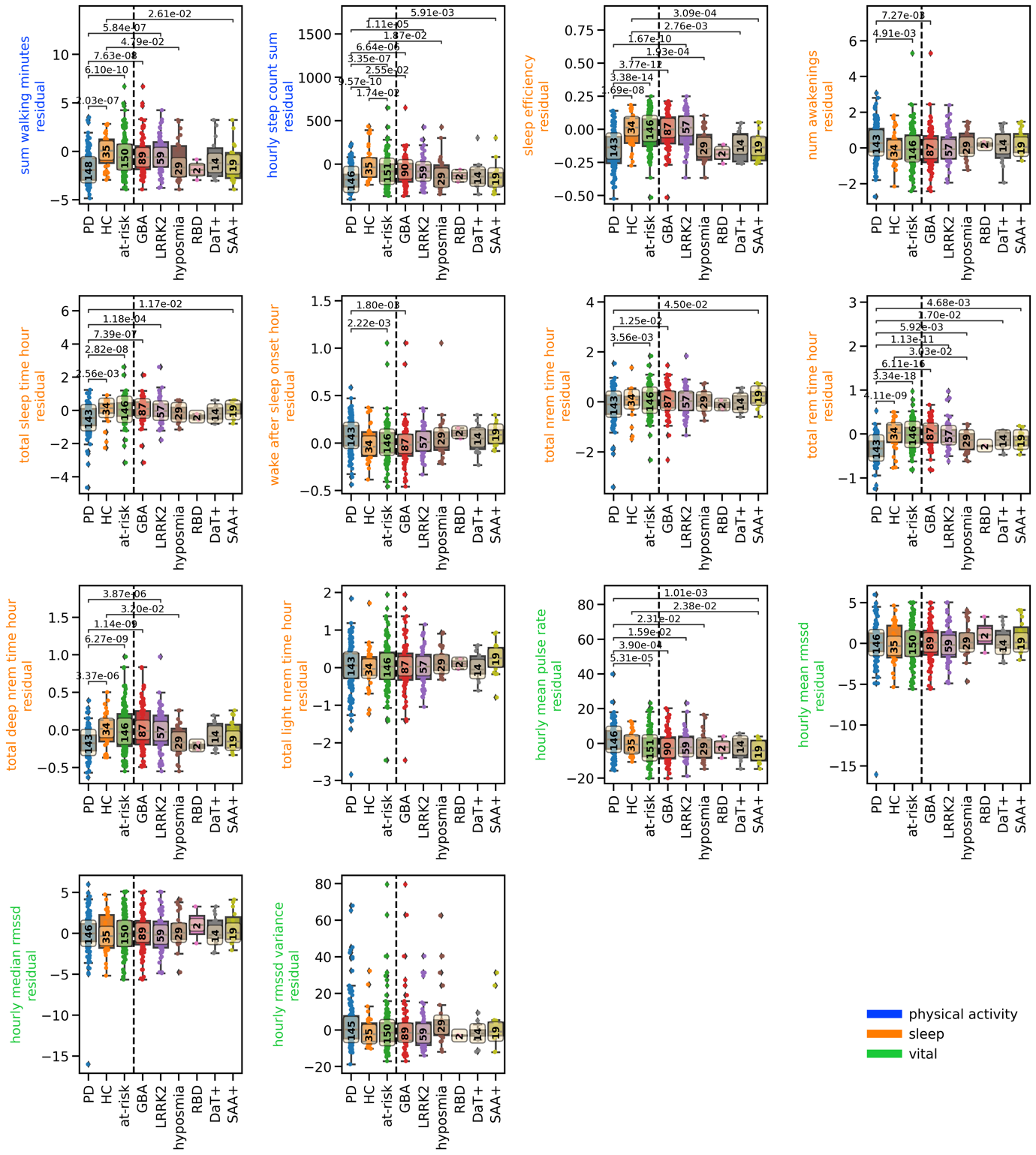


## **Supplementary Figure 1: Mean digital markers are affected in people diagnosed with PD**

The boxplots show the residual overall mean adjusted for age and sex with parameters learned from a linear regression on the healthy controls. The overall mean is computed over the whole observation time per subject for each group for each digital marker. The boxplots depict the group median and quartiles per group with the whiskers showing the Q3+1.5 interquartile range (IQR) and Q1-1.5 IQR (Parkinson’s disease cases: PD; healthy controls: HC; carriers of genetic risk alleles or prodromal symptoms without a diagnosis of PD: *GBA, SNCA, LRRK2*, olfactory loss, PSG-proven RBD, positive DaTscan, positive SAA, union of these: at-risk). The number in the yellow box indicates the number of individuals per group. Group differences were calculated with two-sided T-test between PD and HC to each of the prodromal groups. Asterisks show significant differences after 0.05 Bonferroni-correction.


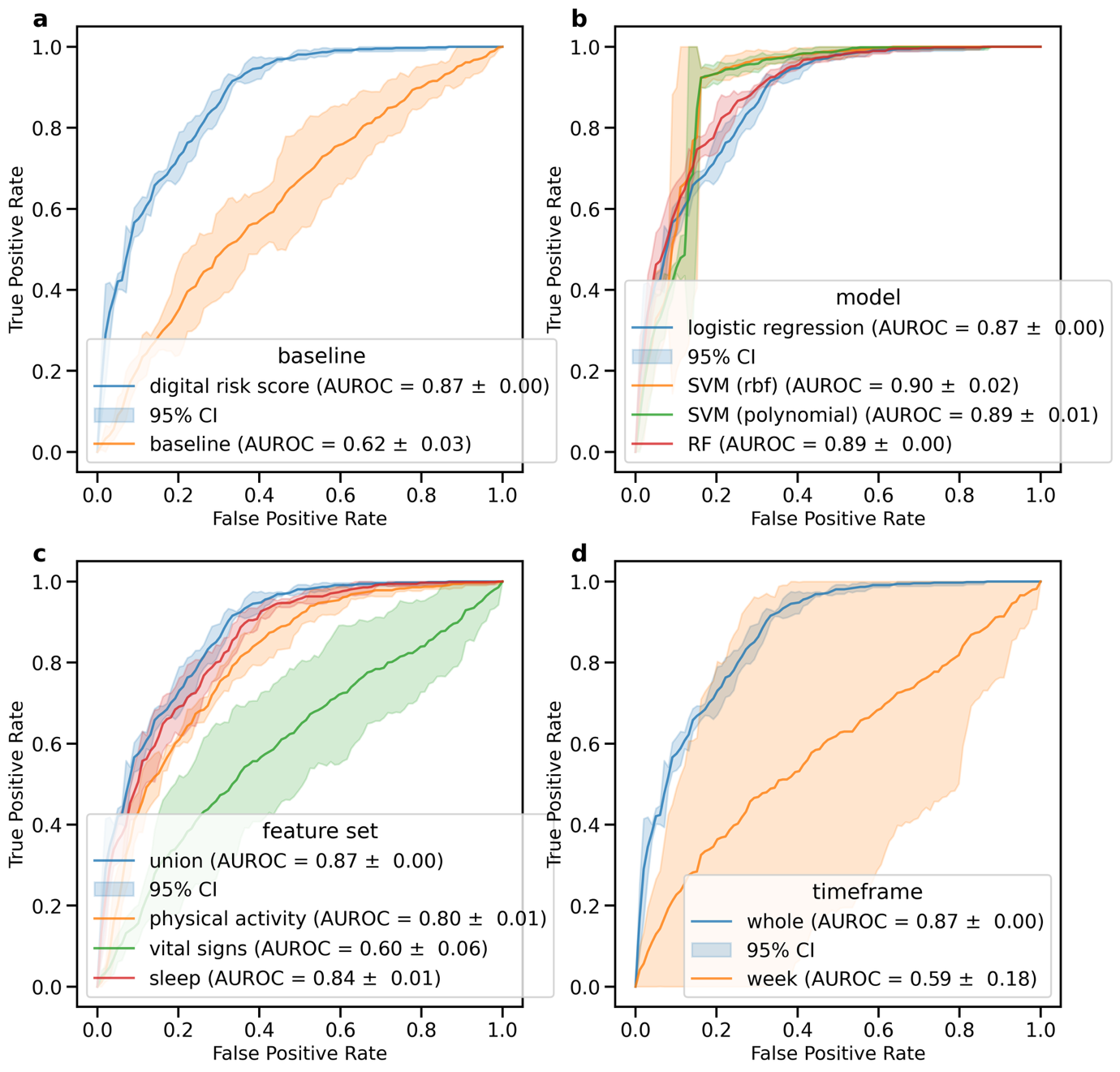


## **Supplementary Figure 2: Performance of digital risk models in terms of area under receiver operator curve.** The performances for the digital risk score models are shown compared to a) baseline, b) other machine learning models, c) other feature sets, and d) other considered time frames. The area under receiver operator curves are shown as the mean on the outer 5-folds of the nested cross-validation. The shaded area displays the 95% Confidence Interval (CI). For each classifier, the legend shows the mean area under receiver operator curve with the standard deviation.


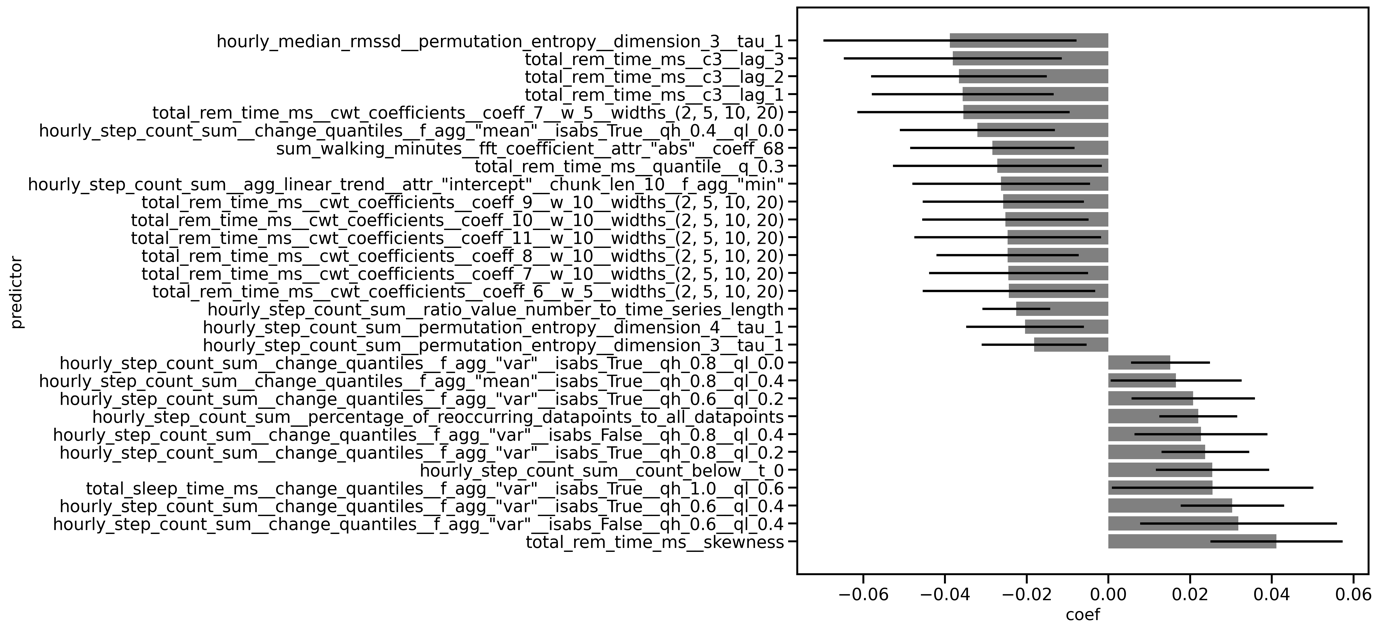


## **Supplementary Figure 3: Significant predictors of digital risk model.** The predictors consistently and significantly selected across folds are shown with their mean effect size across folds and the 95% Bonferroni-corrected Confidence Interval. Significance across folds was determined with a one-sample ttest of the coefficients across folds with 0.05 Bonferroni-corrected significance level.

**
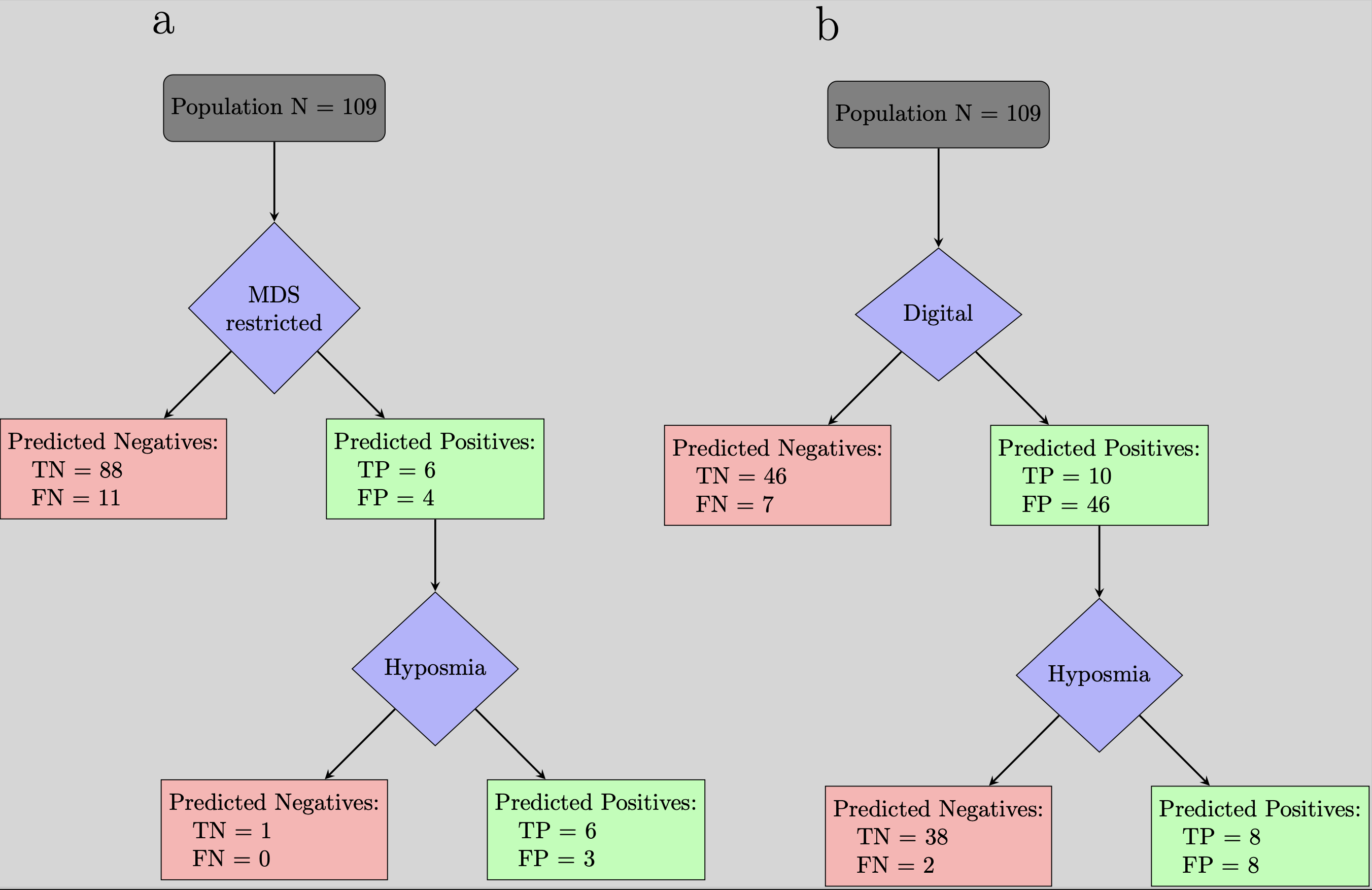
**

## **Supplementary Figure 4: Digital risk score as a sensitive screening tool in a sequence of testing**

The flowcharts displays a potential chaining of tests to identify people at risk of Parkinson’s disease. Here the tests are evaluated against presence of biological (DaTscan) or pathological (alpha-synuclein SAA) markers due to a lack of future phenoconversion information.

a) Starting with the restricted MDS prodromal risk as defined by Heinzel, Berg, Gasser, Chen, Yao, Postuma and Disease 2, 10 people are sent for further examination of hyposmia.

b) Starting with the most sensitive and accessible test, the digital risk score, 56 of the 109 individuals are sent for further testing to evaluate hyposmia which increases the specificity.
